# Effects of Zinc Supplementation on Glycemic Control, Insulin Resistance, Inflammation and Oxidative Stress in Diabetes: A Systematic Review and Meta‐Analysis of Randomized Controlled Trials

**DOI:** 10.1002/edm2.70264

**Published:** 2026-08-13

**Authors:** Jessica Paola Loaiza‐Giraldo, Florencia Amigo‐Fierro, Camila Ignacia Cancino‐Castro, Matias Donoso‐Emig, Murtaja Satea, Ignacia Farías‐Quinteros, Alejandro Bruna‐Mejias, Pablo Nova‐Baeza, Mathias Orellana‐Donoso, Héctor Gutiérrez‐Espinoza, Gloria Cifuentes‐Suazo, Juan Sanchis‐Gimeno, Maria Piagkou, Vitor E. Valenti, Jose E. Leon‐Rojas, Juan José Valenzuela‐Fuenzalida

**Affiliations:** ^1^ Facultad de Ciencias de la Salud Universidad Central del Valle del Cauca (UCEVA) Tuluá Valle del Cauca Colombia; ^2^ Departamento de Morfología, Facultad de Medicina Universidad Andrés Bello Santiago Chile; ^3^ Department of Internal Medicine College of Medicine, University of Warith Al‐Anbiyaa Karbala Iraq; ^4^ Escuela de Medicina, Universidad Finis Terrae Santiago Chile; ^5^ Faculty of Medicine and Science Universidad San Sebastián Santiago Chile; ^6^ Faculty of Education Universidad Autónoma de Chile Santiago Chile; ^7^ Universidad Espíritu Santo Samborondón Ecuador; ^8^ Facultad de Medicina, Carrera de Odontología Universidad Católica de la Santísima Concepción Concepción Chile; ^9^ GIAVAL Research Group, Faculty of Medicine, Department of Anatomy and Human Embryology University of Valencia Valencia Spain; ^10^ Department of Anatomy, Faculty of Health Sciences, School of Medicine National and Kapodistrian University of Athens Athens Greece; ^11^ Systematic Reviews Center for Cardiovascular and Metabolic Health, São Paulo State University, School of Philosophy and Sciences Marília São Paulo Brazil; ^12^ Investigacion Bienestar, Salud y Sociedad, Escuela de Psicologia y Educacion, Universidad de Las Americas Quito Ecuador; ^13^ Escuela de Medicina Universidad de Las Américas Quito Ecuador; ^14^ Departamento de Ciencias Química y Biológicas, Facultad de Ciencias de la Salud Universidad Bernardo O'Higgins Santiago Chile

**Keywords:** diabetes mellitus, inflammation, insulin resistance, oxidative stress, randomized controlled trials, type 2 diabetes, zinc supplementation

## Abstract

**Objectives:**

Diabetes mellitus (DM) is a chronic metabolic disorder characterized by insulin resistance, impaired insulin secretion, and increased cardiometabolic and inflammatory burden. Zinc plays a key biological role in insulin synthesis, storage, signalling and antioxidant defence; however, the clinical relevance and consistency of zinc supplementation effects in diabetes remain uncertain.

**Methods:**

A systematic search of PubMed/MEDLINE, Web of Science, Scopus, CINAHL and Google Scholar was conducted to identify randomized controlled trials (RCTs) assessing zinc supplementation in individuals with diabetes, gestational diabetes or prediabetes.

**Results:**

Eighteen RCTs involving 1023 participants met the eligibility criteria. Zinc supplementation significantly increased plasma zinc concentrations (MD = 7.80; 95% CI 4.33 to 11.26) and improved insulin resistance, as reflected by reductions in serum insulin (MD = −2.50; 95% CI −4.69 to −0.31) and HOMA‐IR (MD = −1.10; 95% CI −2.05 to −0.15). Total cholesterol decreased modestly but significantly (MD = −5.70; 95% CI −7.50 to −3.89), representing a relatively small absolute reduction, while LDL cholesterol showed a modest increase (MD = 3.46; 95% CI 1.48 to 5.43), although the clinical relevance of this finding remains uncertain. Inflammatory and oxidative stress markers improved, including reductions in C‐reactive protein (SMD = −0.91; 95% CI −1.43 to −0.38) and malondialdehyde (SMD = −0.76; 95% CI −1.34 to −0.18), alongside an increase in total antioxidant capacity (SMD = 1.79; 95% CI 0.68 to 2.91).

**Conclusions:**

Zinc supplementation was associated with improvements in insulin resistance, inflammatory status and oxidative stress markers in individuals with diabetes.

**Trial Registration:**

International Prospective Register of Systematic Reviews (PROSPERO) registration number: CRD42025638646

## Graphical abstract

Zinc supplementation in diabetes is associated with improved insulin sensitivity, lipid profile, antioxidant status and reduced inflammation, without significant effects on glycemic indices. Overall, evidence from randomized trials supports zinc as a beneficial metabolic adjunct with moderate‐to‐high certainty.

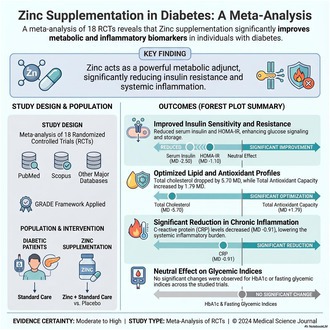

## 1 Introduction

Diabetes mellitus (DM) is a major chronic metabolic disorder affecting millions of individuals worldwide, with global prevalence projected to reach 578 million cases by 2030 [1]. In parallel with its rising incidence, DM imposes a substantial economic burden; in the United States, average annual medical expenditures per patient with diabetes are estimated at $12,022 [2]. The disease is characterized by persistent hyperglycemia resulting from impaired insulin secretion, reduced insulin sensitivity or both. Clinically, DM includes type 1 diabetes mellitus (T1DM), caused by autoimmune destruction of pancreatic β‐cells; type 2 diabetes mellitus (T2DM), driven primarily by insulin resistance with progressive β‐cell dysfunction; and gestational diabetes mellitus (GDM), which develops during pregnancy and is associated with increased long‐term cardiometabolic risk [3].

Zinc is an essential trace element required for normal cellular growth, metabolic regulation, immune function and reproductive health [4]. It is abundant in protein‐rich foods such as meat, fish and legumes and is widely available as a dietary supplement. Zinc is the second most abundant trace mineral in the human body after iron [5]. Absorption occurs mainly in the small intestine via zinc transporters such as ZIP4, after which zinc circulates primarily bound to albumin and is distributed to peripheral tissues [6]. Zinc serves as a catalytic or structural cofactor for more than 300 enzymes involved in DNA synthesis, immune modulation, antioxidant defence and cellular repair [6, 7]. Intracellular zinc availability is tightly regulated by metallothioneins, which buffer zinc levels and protect cells from oxidative damage [8]. Excess zinc is primarily eliminated through intestinal and pancreatic secretions, thereby maintaining physiological homeostasis. Optimal serum zinc concentrations (12–16 μM) support immune defence, wound healing, synaptic plasticity and membrane stability [6, 9].

Increasing evidence supports a close association between zinc homeostasis and the pathophysiology of diabetes. Zinc plays a direct role in pancreatic β‐cell function by facilitating insulin synthesis, crystallization, storage and regulated exocytosis [6, 7]. Zinc deficiency has been linked to impaired insulin signalling, increased oxidative stress and heightened inflammatory response mechanisms central to the development and progression of insulin resistance and T2DM [8, 10]. Conversely, adequate zinc status has been associated with improved glycemic control and a reduced risk of diabetes onset [10]. In addition to its metabolic effects, zinc contributes to antioxidant defence by stabilizing enzymes involved in detoxifying reactive oxygen species, potentially mitigating oxidative damage induced by chronic hyperglycemia.

Despite strong biological plausibility, clinical trials evaluating zinc supplementation in individuals with diabetes have produced inconsistent findings. Reported effects on glycemic control, lipid profiles, inflammation and oxidative stress vary widely across studies, likely reflecting differences in diabetes phenotype, baseline zinc status, dose, formulation, intervention duration and co‐supplementation with other micronutrients. As a result, the magnitude, consistency and clinical relevance of zinc's effects in diabetic populations remain uncertain.

Therefore, the objective of this systematic review and meta‐analysis was to evaluate the effects of zinc supplementation on biochemical, metabolic, inflammatory and oxidative stress outcomes in individuals with diabetes, gestational diabetes or prediabetes, and to assess the certainty of evidence supporting its potential role as an adjunctive nutritional strategy in diabetes management.

## 2 Methods

### 2.1 Protocol and Registration

This systematic review and meta‐analysis was conducted and reported in accordance with the Preferred Reporting Items for Systematic Reviews and Meta‐Analyses (PRISMA) guidelines [11]. The study protocol was prospectively registered in the International Prospective Register of Systematic Reviews (PROSPERO) under registration number CRD42025638646.

### 2.2 Literature Search

A comprehensive literature search was performed across MEDLINE (via PubMed), EMBASE, Scopus, Web of Science, the Cochrane Central Register of Controlled Trials (CENTRAL) and the Cumulative Index to Nursing and Allied Health Literature (CINAHL). Searches included all records from database inception through January 2026. Randomized or controlled clinical trials published in English or Spanish were considered eligible. The search strategy combined controlled vocabulary and free‐text terms using Boolean operators. Key terms included ‘diabetes mellitus’, ‘type 1 diabetes mellitus’, ‘type 2 diabetes mellitus’ and ‘zinc supplementation’. Two reviewers (J.J.V.‐F. and Fa) independently screened titles and abstracts to identify potentially eligible studies. Full‐text articles were retrieved when eligibility could not be determined from abstracts alone. Discrepancies were resolved by consensus, and unresolved disagreements were adjudicated by a third reviewer (M.B.‐V.) (Table S1).

### 2.3 Study Selection

Studies were eligible if they met the following criteria: (1) enrolled human participants diagnosed with type 1 or type 2 diabetes mellitus; (2) evaluated zinc supplementation at any dose or duration; (3) reported biochemical, haematological, metabolic or clinical outcomes; and (4) employed a randomized or controlled experimental design with a comparator group. Eligible designs included randomized clinical trials and controlled clinical trials.

Studies were excluded if they: (1) were non‐original articles (e.g., reviews, editorials, case reports or case series); (2) involved animal or in vitro models; (3) enrolled participants with comorbid conditions unrelated to diabetes; (4) evaluated interventions other than zinc supplementation without an isolated zinc arm; or (5) lacked a concurrent control group.

Trials evaluating zinc in combination with other micronutrients or bioactive compounds were included only when zinc was absent from the control arm and the study design allowed comparison against placebo or standard care. However, because co‐interventions may independently influence metabolic and inflammatory outcomes, causal attribution of pooled effects specifically to zinc supplementation remains limited. Therefore, findings derived from multicomponent interventions were interpreted cautiously throughout the review (Table S2).

### 2.4 Data Extraction and Risk of Bias Assessment

Two reviewers (M.B.‐V. and M.L.‐C.) independently extracted data using a standardized data extraction form. Extracted information included author and year of publication, study design, sample size, participant characteristics, intervention details (dose, formulation, route and duration), comparator characteristics, outcomes assessed, statistical measures, primary findings and reported adverse events. Discrepancies were resolved through discussion or, when necessary, by consultation with a third reviewer (J.‐L.G.) (Table S3).

Methodological quality and risk of bias were assessed using the Cochrane Risk of Bias tool (RoB 1.0) [12], which evaluates seven domains: random sequence generation, allocation concealment, blinding of participants and personnel, blinding of outcome assessment, incomplete outcome data, selective reporting and other sources of bias. Each domain was classified as low, unclear or high risk of bias. Inter‐reviewer agreement was assessed using Cohen's kappa coefficient, demonstrating substantial agreement (*κ* = 0.91).

### 2.5 Data Synthesis and Statistical Analysis

Outcomes were categorized as biochemical, clinical, inflammatory or oxidative stress parameters and analysed as continuous variables. Effect sizes were expressed as mean differences (MD) when outcomes were reported in the same units and as standardized mean differences (SMD) when different measurement scales or assays were used, each with corresponding 95% confidence intervals (CI).

Pooled analyses were conducted using either the Hartung–Knapp–Sidik–Jonkman random‐effects model or the Mantel–Haenszel fixed‐effects model, depending on the degree of between‐study heterogeneity. Random‐effects models were preferentially applied when substantial or considerable heterogeneity was present. Statistical heterogeneity was quantified using the *I* ^2^ statistic and interpreted as follows: 0%–40% (not important), 30%–60% (moderate), 50%–90% (substantial) and 75%–100% (considerable). Consistency across studies was further assessed by visual inspection of forest plots and by the overlap of confidence intervals.

Prespecified subgroup analyses were conducted according to intervention duration. Meta‐regression analyses were performed to evaluate the influence of predefined moderators, including mean age, sex distribution, supplementation duration and daily zinc dose. All analyses were conducted using Review Manager (RevMan) version 5.4 (The Cochrane Collaboration). Clinical interpretation of pooled results was based on whether higher or lower values represented favourable physiological effects rather than statistical direction alone.

Given the anticipated clinical and methodological heterogeneity across diabetes phenotypes, intervention protocols, and participant characteristics, random‐effects models were preferentially applied for outcomes demonstrating substantial between‐study variability. Consequently, pooled estimates should be interpreted as average effects across heterogeneous populations rather than precise estimates applicable to individual clinical settings.

### 2.6 Clinical Reference Ranges and Outcome Interpretation

Biochemical, clinical, inflammatory and oxidative stress outcomes were interpreted according to internationally accepted clinical reference ranges. Normal serum zinc concentrations were defined as 70–120 μg/dL (10.7–18.4 μmol/L). Normal fasting plasma glucose (FPG) and fasting blood glucose (FBG) were defined as 70–99 mg/dL, while serum insulin concentrations between 2 and 20 μU/mL were considered normal.

Insulin resistance and β‐cell function were assessed using the Homeostatic Model Assessment (HOMA) indices; HOMA‐IR < 2.7 indicates normal insulin sensitivity, and HOMA‐B values between 60% and 100% reflect normal β‐cell function. Lipid parameters were interpreted according to standard clinical guidelines: triglycerides < 150 mg/dL; VLDL cholesterol 5–40 mg/dL; total cholesterol < 200 mg/dL; LDL cholesterol < 100 mg/dL (optimal), 100–129 mg/dL (acceptable) and ≥ 160 mg/dL (high); and HDL cholesterol > 40 mg/dL in men and > 50 mg/dL in women. The total cholesterol/HDL cholesterol ratio was classified as ideal (< 3.5), acceptable (3.5–5.0), or high risk (> 5.0).

Glycemic control was further evaluated using glycated haemoglobin (HbA1c), with values < 5.7% considered normal, 5.7%–6.4% indicating prediabetes and ≥ 6.5% consistent with diabetes. Serum magnesium levels of 1.7–2.2 mg/dL (0.70–0.95 mmol/L) were considered normal. Insulin sensitivity was additionally assessed using the Quantitative Insulin Sensitivity Check Index (QUICKI), with normal values ranging from 0.357 to 0.450. Clinical outcomes included systolic blood pressure (SBP) (normal < 120 mmHg) and body mass index (BMI) (normal 18.5–24.9 kg/m^2^).

Inflammatory status was assessed using C‐reactive protein (CRP) or high‐sensitivity CRP (hs‐CRP), with values < 10 mg/L considered normal. Oxidative stress and antioxidant capacity were evaluated using malondialdehyde (MDA) (1–3 μmol/L), nitric oxide (NO) (20–40 μmol/L), total antioxidant capacity (TAC) (1–1.5 mmol Trolox/L) and total glutathione (GSH) (450–600 μmol/L or 7–10 mg/dL). These reference ranges were applied to standardize outcome interpretation across studies.

### 2.7 Certainty of Evidence Assessment

The certainty of evidence for each outcome was evaluated using the Grading of Recommendations, Assessment, Development and Evaluation (GRADE) approach [13]. Evidence was classified as high, moderate, low or very low based on risk of bias, inconsistency, indirectness, imprecision and publication bias. GRADEpro GDT software was used to import effect estimates from RevMan 5.4 and generate the Summary of Findings table. Detailed GRADE assessments are presented in (Table S4).

## 3 Results

### 3.1 Study Selection

**FIGURE 1:**
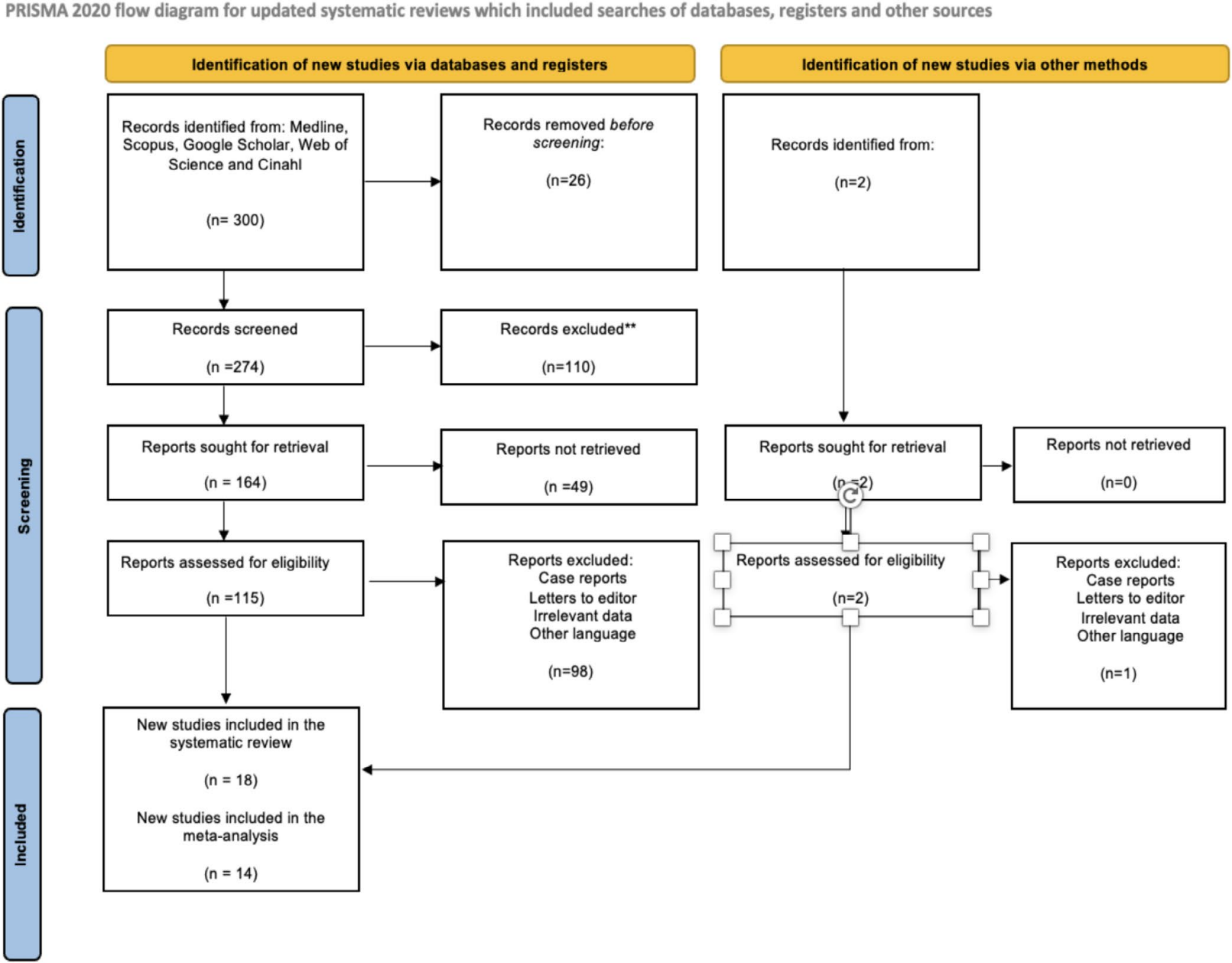
Flow diagram.

The electronic search identified 300 records. After removal of duplicates and screening of titles and abstracts, 115 articles were retrieved for full‐text evaluation. The selection process is presented in the PRISMA flow diagram (Figure 1). No additional eligible trials were identified through clinical trial registries. Overall, 18 randomized controlled trials (RCTs) met the inclusion criteria and were included in the systematic review and meta‐analysis [11, 12, 13, 14, 15, 16, 17, 18, 19, 20, 21, 22, 23, 24, 25, 26, 27, 28].

### 3.2 Study Characteristics

Characteristics of included studies are summarized in Table 1. The 18 trials compared various zinc supplementation regimens with placebo or standard care controls [11, 12, 13, 14, 15, 16, 17, 18, 19, 20, 21, 22, 23, 24, 25, 26, 27, 28]. Studies were conducted in Iran, Tunisia, Australia, Chile, Egypt, Bangladesh, the United Kingdom and Singapore, and were published between 2001 and 2022. A total of 1023 participants were included (510 allocated to zinc supplementation and 513 to the control group). Mean age was 43.76 ± 5.82 years in the zinc groups and 44.27 ± 5.53 years in the control groups. Trials by Reza and Pérez were excluded from the age averaging process because age was reported as ranges or not explicitly provided [24, 27]. The mean supplementation duration was 19.39 weeks (range: 6–52 weeks).

**TABLE 1:** Characteristics of studies.

Author, year	Country	Population	*N* (IG/CG)	Zinc dose (mg/day, elemental)	Duration (weeks)	Primary outcomes
Karamali et al., 2015	Iran	GDM	29/29	30 mg	6	FPG, Insulin, HOMA‐IR, HOMA‐B
Anderson et al., 2001	Tunisia	T2DM	27/29	30 mg	26	FPG, HbA1c, Insulin
Asghari et al., 2019	Iran	T2DM	30/30	30 mg	12	FBG, HbA1c, Insulin, HOMA‐IR
Hamedifard et al., 2020	Iran	T2DM + CHD	27/28	30 mg	12	FPG, Insulin, HOMA‐IR
Jamilian et al., 2019	Iran	GDM	30/30	4 mg	6	FPG
Attia et al., 2022	Australia	Prediabetes	48/50	30 mg	52	FBG, HbA1c, HOMA‐IR
Nazem et al., 2022	Iran	T2DM	35/35	50 mg	8	FBG, HbA1c, Insulin, HOMA‐IR
Pérez et al., 2018	Chile	T2DM	13/15	30 mg	52	FBG, HbA1c, HOMA‐IR
Matter et al., 2019	Egypt	β‐TM + DM (paediatric)	38/39	40 mg	12	FBG, HOMA‐IR
Momen‐Heravi et al., 2017	Iran	DFU + T2DM	30/30	50 mg	12	FPG, HbA1c, Insulin, HOMA‐IR
Karamali et al., 2016	Iran	GDM	25/25	30 mg	6	hs‐CRP, TAC, MDA, NO, GSH, Zinc
Karandish et al., 2021	Iran	Prediabetes (overweight)	21/21	30 mg	12	FPG, HbA1c, Insulin
Nazem et al., 2019	Iran	T2DM (overweight)	35/35	50 mg	8	FBG, HbA1c, Insulin, HOMA‐IR
Shidfar et al., 2010	Iran	T1DM	25/23	10 mg	13	FBG, Insulin
Islam et al., 2016	Bangladesh	Prediabetes	28/27	30 mg	26	FBG, Insulin, HOMA‐IR
Hosseini et al., 2022	Iran	T2DM + Zn‐deficiency	21/22	50 mg	8	FBG, Insulin, HOMA‐IR
Roussel et al., 2003	United Kingdom	T2DM	54/54	30 mg	26	FPG, HbA1c, Insulin
Seet et al., 2011	Singapore	T2DM	20/20	75 mg	13	Oxidative stress biomarkers (F_2_‐IsoPs, HETEs, neuroprostanes, oxysterols), vascular function (PWV, AIx, BP)

Abbreviations: β‐TM, beta‐thalassaemia major; CHD, coronary heart disease; DFU, diabetic foot ulcer; FBG, fasting blood glucose; FPG, fasting plasma glucose; GDM, gestational diabetes mellitus; HbA1c, glycated haemoglobin; HOMA‐IR, homeostatic model assessment‐insulin resistance; T1DM, type 1 diabetes mellitus; T2DM, type 2 diabetes mellitus.

### 3.3 Other Treatment Characteristics

Several trials evaluated zinc as part of multicomponent interventions, limiting the attribution of observed effects to zinc alone. These studies were retained because zinc was included in the intervention arm and compared with placebo or standard care; however, findings were interpreted cautiously.

Zinc plus magnesium. Two studies assessed the combination of zinc and magnesium. Hamedifard et al. [16] evaluated zinc plus magnesium in patients with T2DM and coronary heart disease and reported reductions in fasting plasma glucose and serum insulin compared with placebo. Although HOMA‐IR did not reach statistical significance, the overall pattern suggested improved insulin sensitivity. Co‐supplementation also increased HDL‐cholesterol and reduced CRP and MDA, consistent with anti‐inflammatory and antioxidative effects [16]. Jamilian et al. [12] studied zinc, magnesium, calcium and vitamin D in women with gestational diabetes and reported reductions in fasting plasma glucose and improvements in antioxidant markers (higher TAC and lower MDA), without significant effects on HbA1c, serum insulin, or HOMA‐IR; the contribution of zinc could not be isolated.

Zinc plus curcumin. Karandish et al. [20] examined zinc co‐administered with curcumin for 90 days in individuals with T2DM and reported reductions in fasting plasma glucose, HbA1c and insulin resistance markers, with improved insulin sensitivity; causal attribution to zinc remains uncertain due to curcumin's known metabolic effects.

Zinc plus vitamin Shidfar et al. evaluated zinc combined with vitamin A in individuals with type 1 diabetes and reported reductions in serum insulin and the apolipoprotein B/A‐I ratio, suggesting potential improvements in insulin metabolism and cardiovascular risk markers. Fasting blood glucose was not explicitly reported, and the dual‐supplement design precludes isolating zinc‐specific effects [26].

### 3.4 Risk of Bias Assessment

**FIGURE 2:**
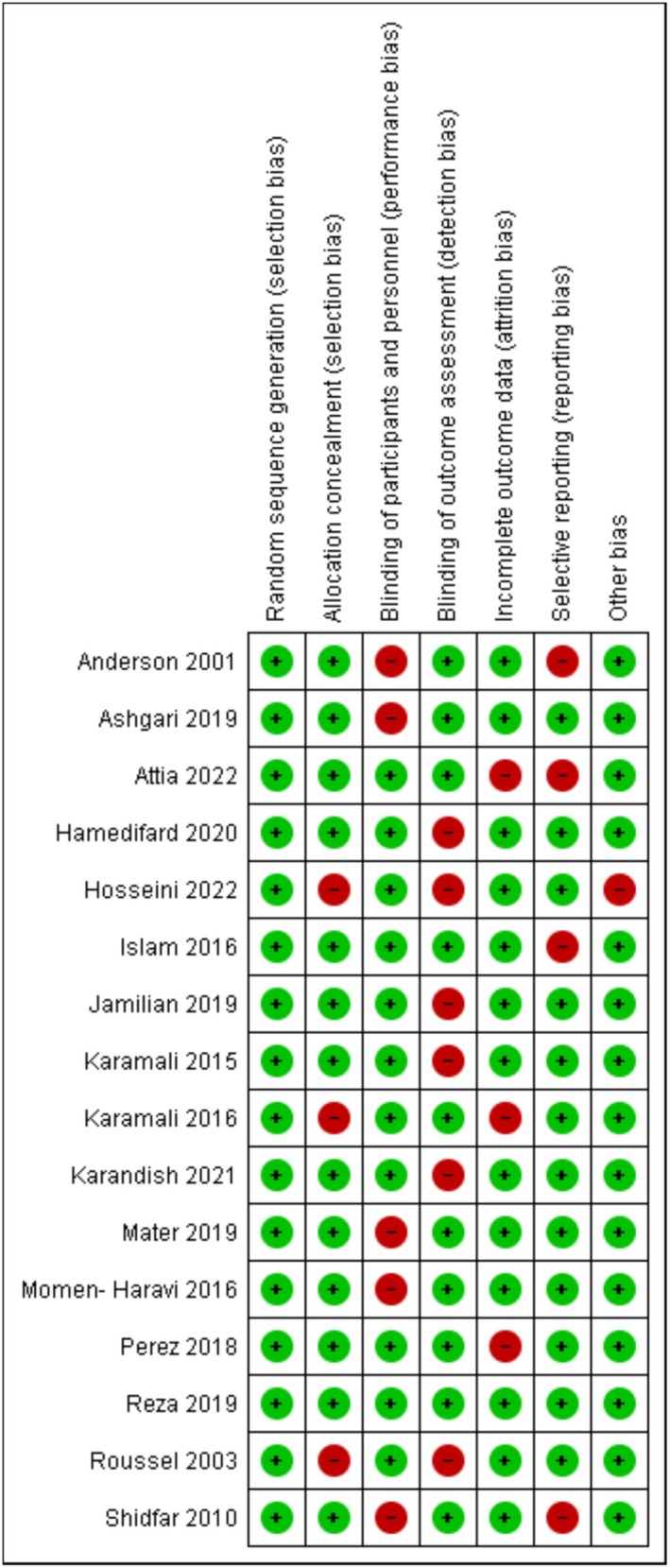
Risk of bias summary included studies [17, 18, 19, 20, 21, 22, 23, 24, 25, 26, 27, 28, 29, 30, 31].

**FIGURE 3:**
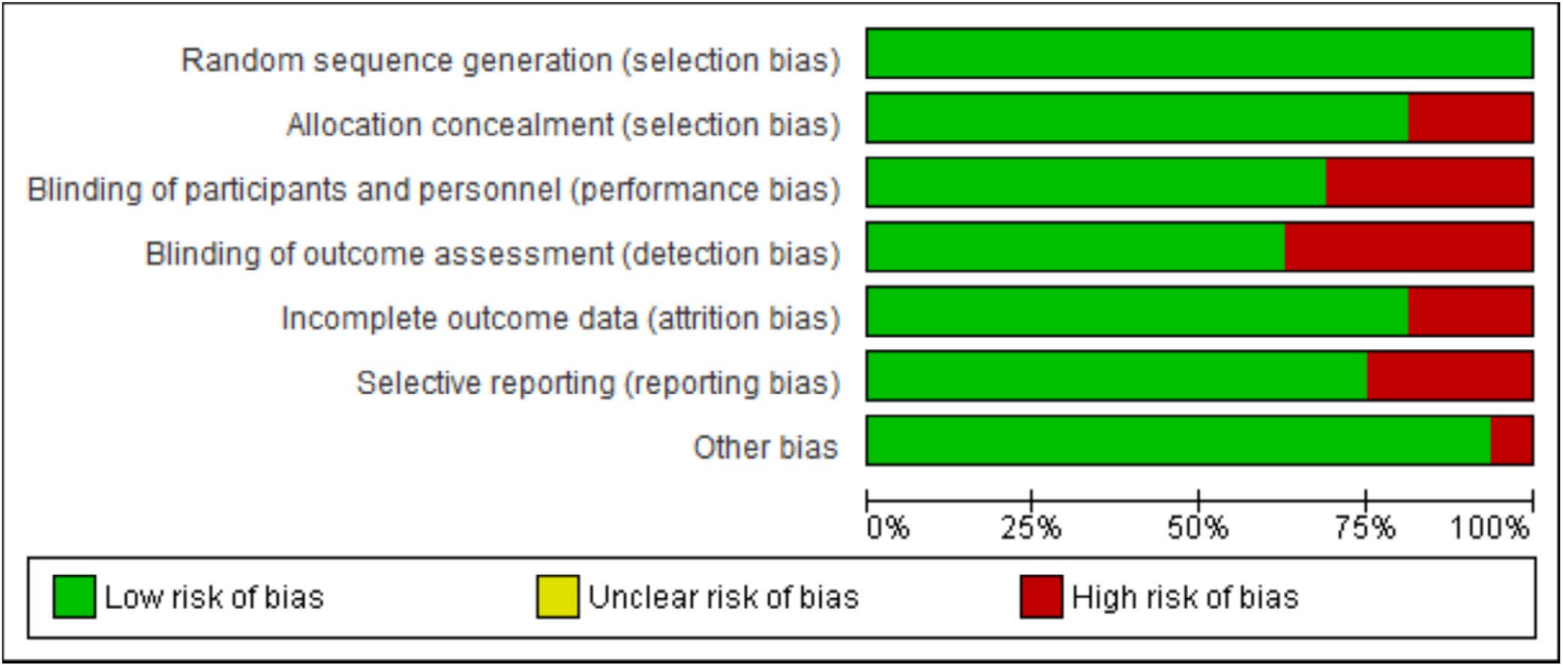
Diagram risk of bias of the studies included.

Risk of bias was assessed using the Cochrane Risk of Bias tool (RoB 1.0) and is summarized in Figures 2 and 3. Random sequence generation was rated as low risk in 93.3% (14/15) of studies and as unclear in 6.7% (1/15) [11, 12, 13, 14, 15, 16, 17, 18, 19, 20, 21, 22, 23, 24, 25]. Allocation concealment was low risk in 26.7% (4/15), high risk in 66.7% (10/15) and unclear in 6.7% (1/15) [11, 12, 13, 14, 15, 16, 17, 18, 19, 20, 21, 22, 23, 24, 25, 26, 27]. Blinding of participants and personnel was low risk in 60% (9/15), unclear in 6.7% (1/15) and high risk in 33.3% (5/15) [11, 12, 13, 14, 15, 16, 17, 18, 19, 20, 21, 22, 23, 24, 25, 26, 27]. Blinding of outcome assessment was low risk in 53.3% (8/15), high risk in 40% (6/15) and unclear in 6.7% (1/15) [11, 12, 13, 14, 15, 16, 17, 18, 19, 20, 21, 22, 23, 24, 25, 26, 27]. Incomplete outcome data were low risk in 73.3% (11/15), high risk in 6.7% (1/15) and unclear in 20% (3/15) [11, 12, 13, 14, 15, 16, 17, 18, 19, 20, 21, 22, 23, 24, 25, 26, 27]. Selective reporting was low risk in 80% (12/15) and high risk in 20% (3/15) [11, 12, 13, 14, 15, 16, 17, 18, 19, 20, 21, 22, 23, 24, 25, 26, 27]. Other sources of bias were low risk in 66.7% (10/15), unclear in 13.3% (2/15) and high risk in 20% (3/15) [11, 12, 13, 14, 15, 16, 17, 18, 19, 20, 21, 22, 23, 24, 25, 26, 27]. Overall, limitations were most common in allocation concealment, blinding and reporting domains and should be considered when interpreting pooled estimates.

### 3.5 Synthesis of Results

All outcomes were analysed as continuous variables in RevMan 5.4. Effect sizes were reported as mean differences (MD) when outcomes were measured in the same units and as standardized mean differences (SMD) when different scales or measurement approaches were used, each with 95% confidence intervals (CI). When SDs were not reported, they were imputed from 95% CIs using: SD = (Upper CI − Lower CI)/3.92 × √*n*, where *n* is the relevant group sample size. Pooled estimates were calculated using inverse‐variance methods under fixed‐ or random‐effects models, depending on heterogeneity quantified by *I* ^2^. For outcomes where lower values are clinically favourable (e.g., insulin resistance and inflammatory markers), negative pooled estimates were interpreted as beneficial. For outcomes where higher values are favourable (e.g., antioxidant capacity), positive pooled estimates were interpreted as beneficial.

#### 3.5.1 Biochemical Parameters

##### 3.5.1.1 Plasma Zinc Concentrations

**FIGURE 4:**
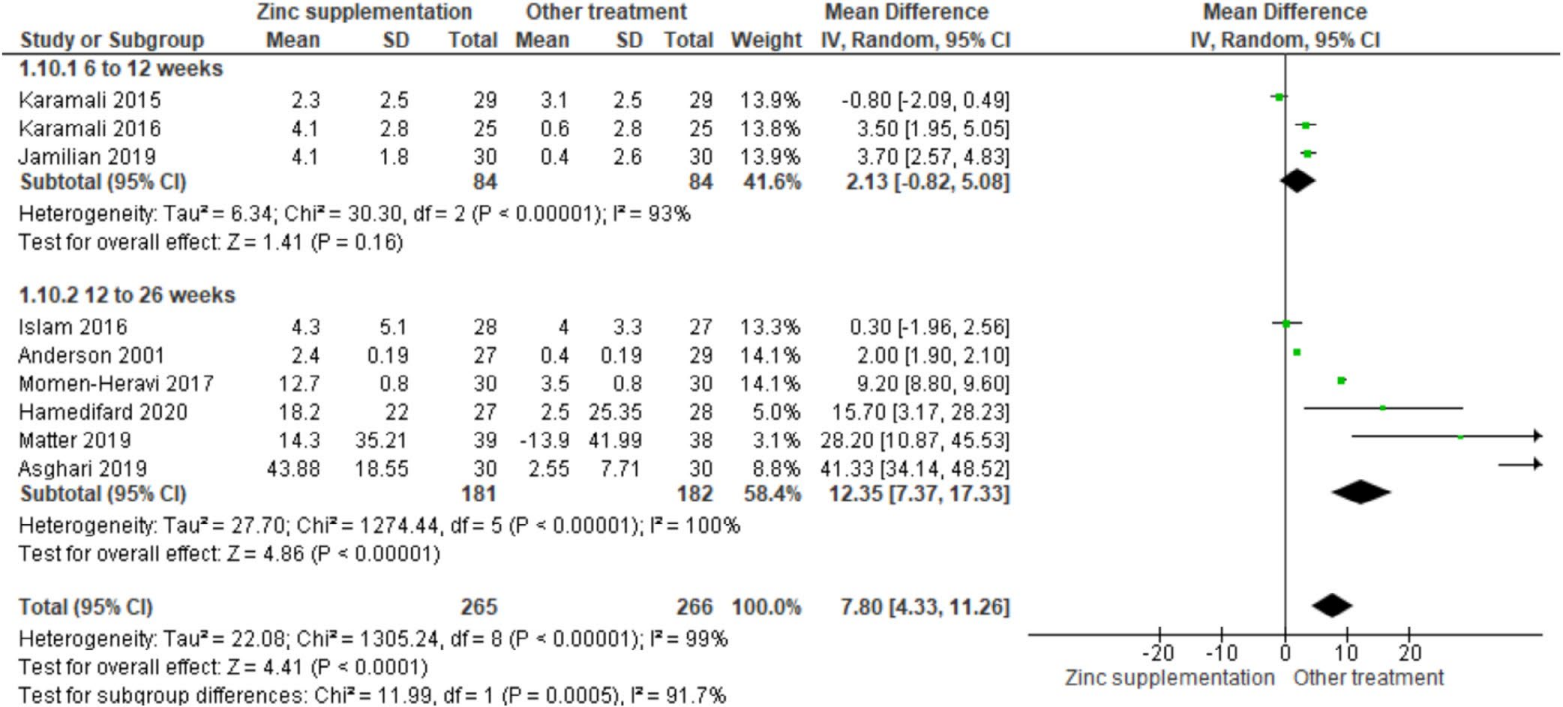
Forest plot plasma zinc.

Overall: Nine studies assessed plasma zinc concentrations (Figure 4). Zinc supplementation significantly increased plasma zinc compared with control [11, 13, 14, 16, 17, 18, 19, 20, 22] (MD = 7.80; 95% CI 4.33 to 11.26; *p* < 0.00001), with considerable heterogeneity (*I* ^2^ = 99%). Certainty of evidence was moderate (Table S4).

6–12 weeks: Three studies (Figure 4) showed no significant difference [12, 13, 17] (MD = 2.13; 95% CI −0.82 to 5.08; *p* = 0.16; *I* ^2^ = 93%).

12–26 weeks: Six studies (Figure 4) showed significantly higher plasma zinc in the zinc group [11, 14, 16, 18, 19, 20] (MD = 12.35; 95% CI 7.37 to 17.33; *p* < 0.00001; *I* ^2^ = 100%).

##### 3.5.1.2 Fasting Plasma Glucose (FPG)

**FIGURE 5:**
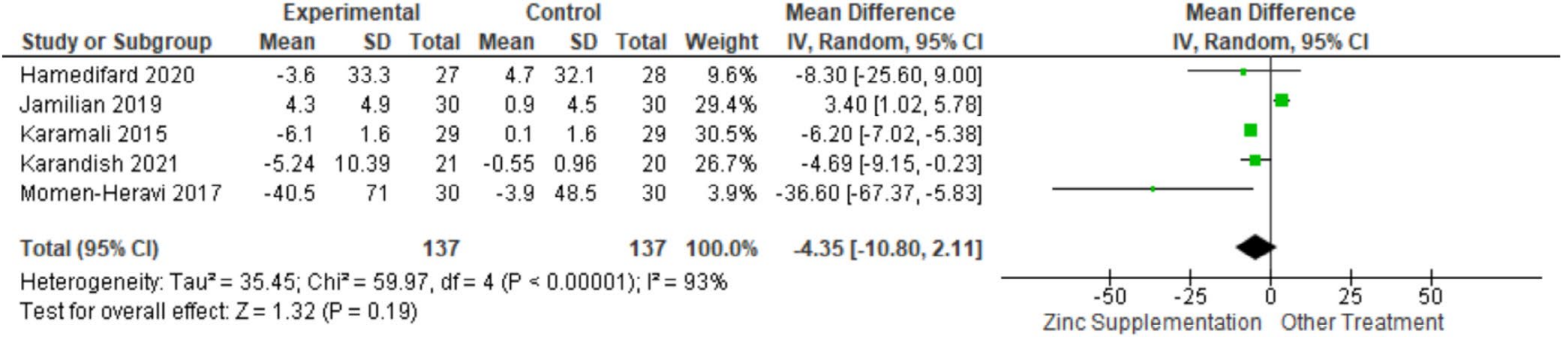
Forest plot fasting plasma glucose (FPG).

Five studies assessed FPG (Figure 5). The available evidence did not demonstrate a significant between‐group difference in FPG [12, 13, 16, 19, 20] (MD = −4.35; 95% CI −10.80 to 2.11; *p* = 0.19), although the limited number of studies and considerable heterogeneity reduce confidence in this null finding. Certainty of evidence was low (Table S4).

##### 3.5.1.3 Fasting Blood Glucose (FBG)

**FIGURE 6:**
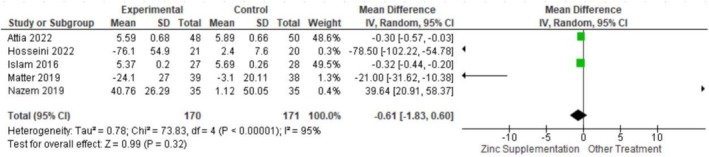
Forest plot fasting blood glucose (FBG).

Five studies assessed FBG (Figure 6). Zinc supplementation did not significantly affect FBG [11, 18, 21, 22, 23] (MD = −0.61; 95% CI −1.83 to 0.60; *p* = 0.32), with considerable heterogeneity (*I* ^2^ = 95%). Certainty of evidence was low (Table S4).

##### 3.5.1.4 Serum Insulin

**FIGURE 7:**
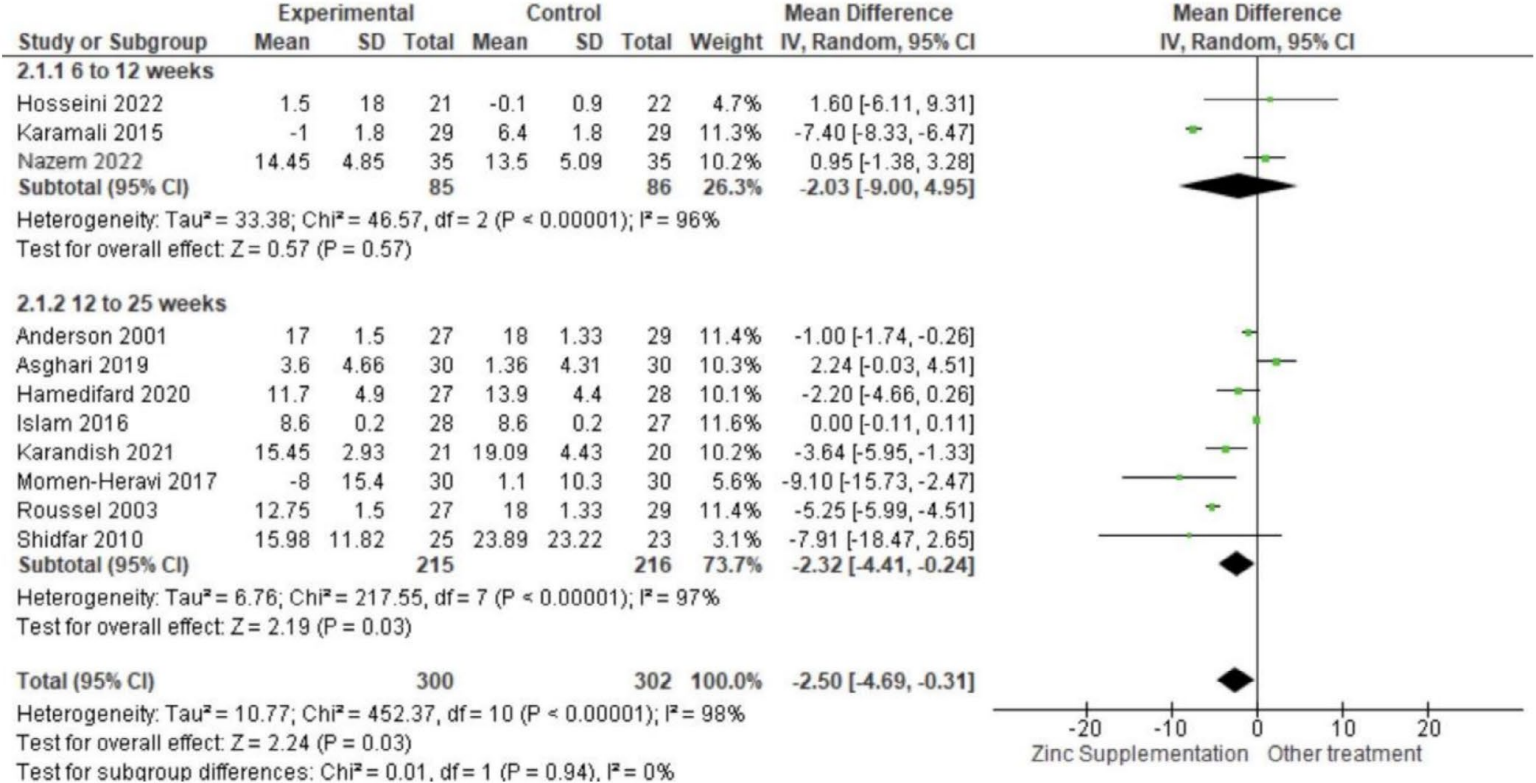
Forest plot serum insulin.

Overall: Eleven studies assessed serum insulin (Figure 7). Zinc supplementation significantly reduced insulin concentrations [11, 13, 14, 15, 16, 19, 20, 22, 23, 25, 26] (MD = −2.50; 95% CI −4.69 to −0.31; *p* = 0.03), with considerable heterogeneity (*I* ^2^ = 98%). Certainty of evidence was moderate (Table S4).

6–12 weeks: Three studies showed no significant differences [13, 22, 23] (MD = −2.03; 95% CI −9.00 to 4.95; *p* = 0.57; *I* ^2^ = 96%).

12–25 weeks: Eight studies showed significantly lower insulin [11, 14, 15, 16, 19, 20, 25, 26] (MD = −2.32; 95% CI −4.41 to −0.24; *p* = 0.03; *I* ^2^ = 97%).

##### 3.5.1.5 HOMA‐IR

**FIGURE 8:**
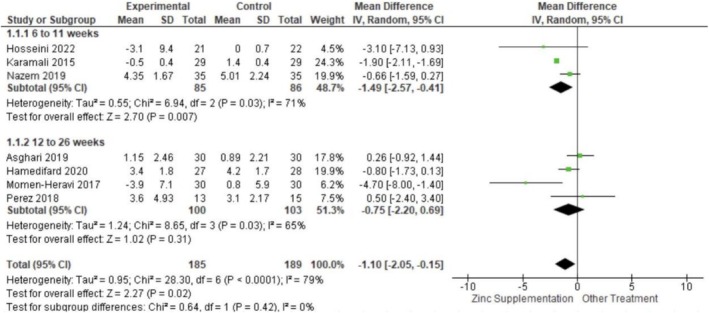
Forest plot HOMA‐IR level.

Overall. Seven studies assessed HOMA‐IR (Figure 8). Zinc supplementation significantly reduced HOMA‐IR [13, 15, 16, 19, 22, 23, 27] (MD = −1.10; 95% CI −2.05 to −0.15; *p* = 0.02), with considerable heterogeneity (*I* ^2^ = 79%). No significant subgroup differences in duration were observed (*p* = 0.42). Certainty of evidence was moderate (Table S4).

6–11 weeks: Three studies showed significant reductions [13, 22, 23] (MD = −1.49; 95% CI −2.57 to −0.41; *p* = 0.007; *I* ^2^ = 71%).

12–26 weeks: Four studies showed no significant differences [15, 16, 19, 27] (MD = −0.75; 95% CI −2.20 to 0.69; *p* = 0.31; *I* ^2^ = 85%).

##### 3.5.1.6 HOMA‐B

**FIGURE 9:**
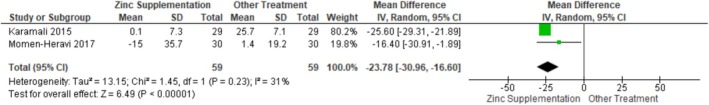
Forest plot HOMA‐B level.

Two studies assessed HOMA‐B (Figure 9). Zinc supplementation was associated with a significant reduction [13, 19] (MD = −23.78; 95% CI −30.96 to −16.60; *p* < 0.00001), with low heterogeneity (*I* ^2^ = 31%). Certainty of evidence was low (Table S4). Given that HOMA‐B reflects β‐cell function, the clinical interpretation of this reduction remains uncertain and may reflect differences in insulin demand or β‐cell activity across included populations.

##### 3.5.1.7 Triglycerides

**FIGURE 10:**
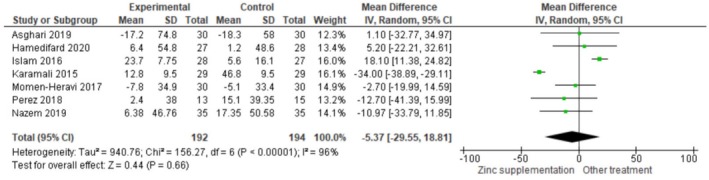
Forest plot triglycerides level.

Seven studies assessed triglycerides (Figure 10). No significant differences were observed [11, 13, 15, 16, 19, 23, 27] (MD = −5.37; 95% CI −29.55 to 18.81; *p* = 0.66), with considerable heterogeneity (*I* ^2^ = 96%). Certainty of evidence was very low (Table S4).

##### 3.5.1.8 Total/HDL Cholesterol Ratio

**FIGURE 11:**
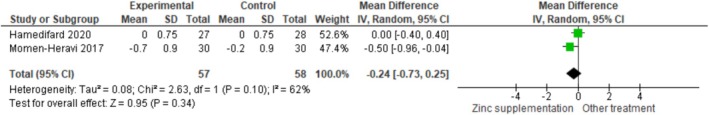
Forest plot total/HDL‐cholesterol ratio.

Two studies assessed the total/HDL cholesterol ratio (Figure 11). No significant effect was observed [16, 19] (MD = −0.24; 95% CI −0.73 to 0.25; *p* = 0.34; *I* ^2^ = 62%). Certainty of evidence was very low (Table S4).

##### 3.5.1.9 VLDL Cholesterol

**FIGURE 12:**
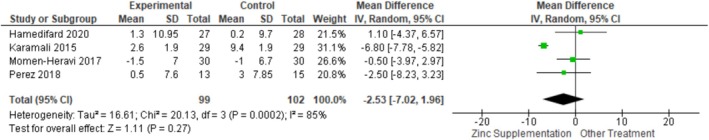
Forest plot VLDL‐cholesterol.

Four studies assessed VLDL cholesterol (Figure 12). No significant differences were observed [13, 16, 19, 27] (MD = −2.53; 95% CI −7.02 to 1.96; *p* = 0.27), with high heterogeneity (*I* ^2^ = 85%). Certainty of evidence was very low (Table S4).

##### 3.5.1.10 Total Cholesterol

**FIGURE 13:**
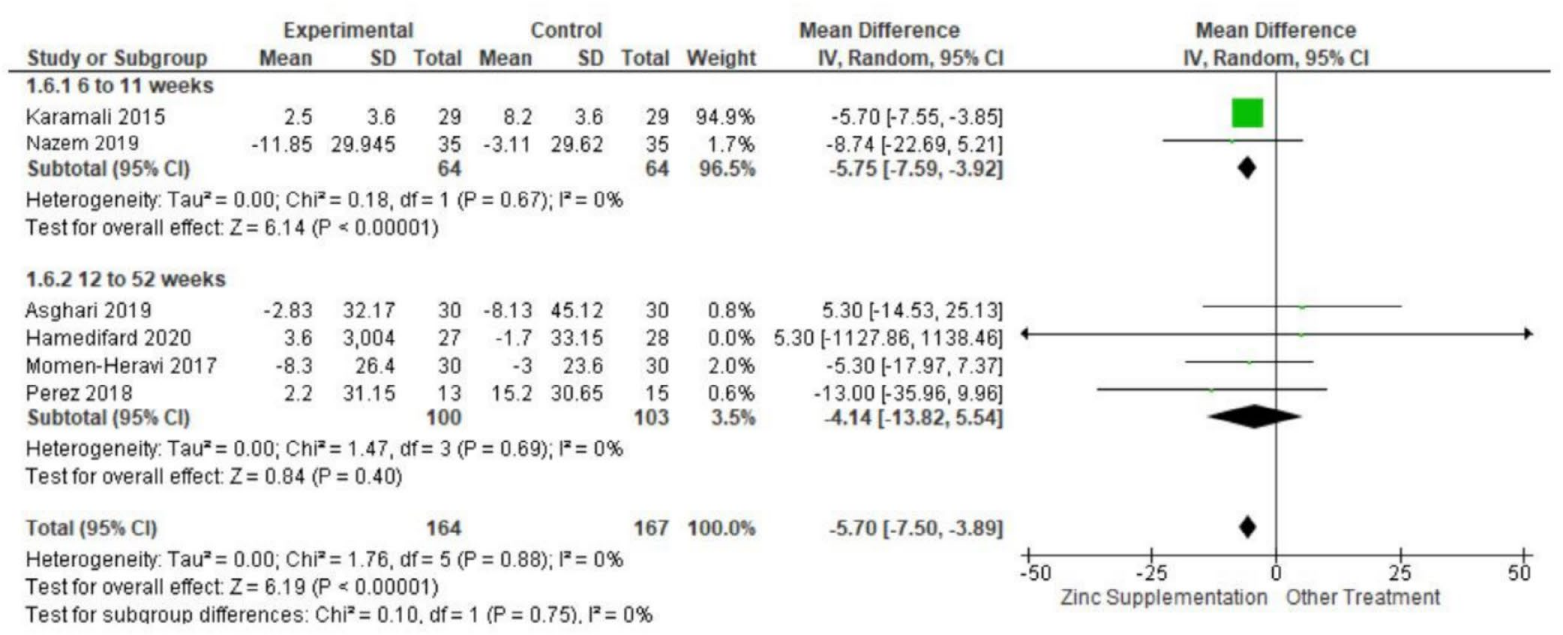
Forest plot total cholesterol.

Six studies demonstrated significantly lower total cholesterol with zinc supplementation (Figure 13) [13, 15, 16, 19, 23, 27] (MD = −5.70; 95% CI −7.50 to −3.89; *p* < 0.00001), with negligible heterogeneity (*I* ^2^ = 0%). The certainty of the evidence was high (Table S4).

6–11 weeks: Two studies showed significant reductions [13, 23] (MD = −5.75; 95% CI −7.59 to −3.92; *p* < 0.00001; *I* ^2^ = 0%).

12–52 weeks: Four studies showed no significant differences [15, 16, 19, 27] (MD = −4.14; 95% CI −13.82 to 5.54; *p* = 0.40; *I* ^2^ = 0%).

##### 3.5.1.11 LDL Cholesterol

**FIGURE 14:**
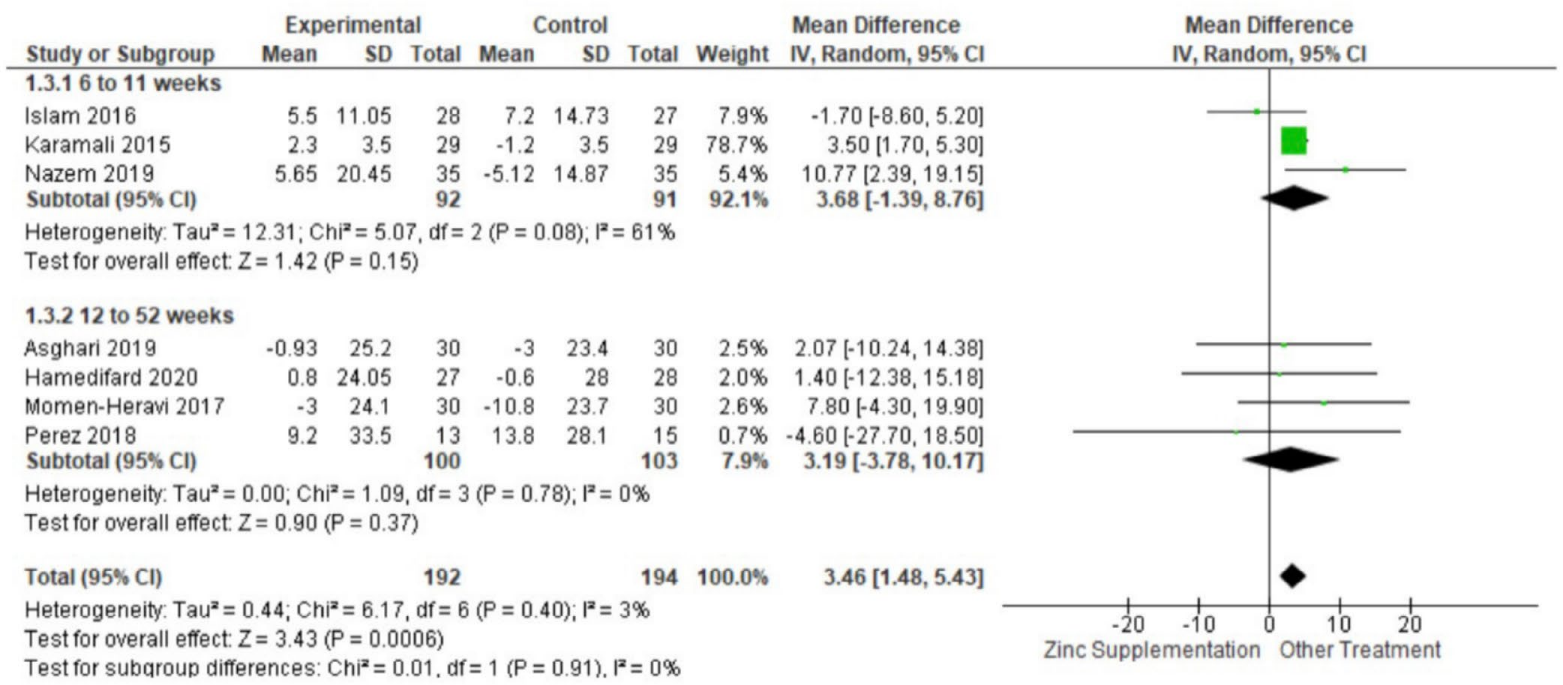
Forest plot LDL‐cholesterol level.

Seven studies demonstrated a significant increase in LDL cholesterol with zinc supplementation (Figure 14) [11, 13, 15, 16, 19, 23, 27] (MD = 3.46; 95% CI 1.48 to 5.43; *p* = 0.0006), with very low heterogeneity (*I* ^2^ = 3%). Certainty of evidence was moderate (Table S4). The clinical significance of this modest increase remains uncertain.

6–11 weeks: Three studies showed no significant differences [11, 13, 23] (MD = 3.68; 95% CI −1.39 to 8.76; *p* = 0.15; *I* ^2^ = 61%).

12–52 weeks: Four studies showed no significant differences [15, 16, 19, 27] (MD = 3.19; 95% CI −3.78 to 10.17; *p* = 0.37; *I* ^2^ = 0%).

##### 3.5.1.12 HDL Cholesterol

**FIGURE 15:**
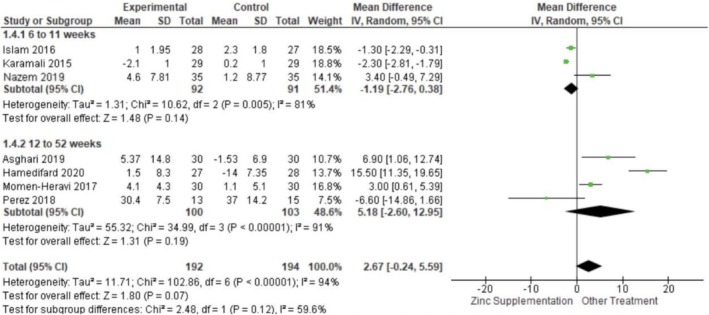
Forest plot HDL‐cholesterol.

Seven studies evaluated HDL cholesterol (Figure 15). No significant differences were observed [11, 13, 15, 16, 19, 23, 27] (MD = 2.67; 95% CI −0.24 to 5.59; *p* = 0.07), with substantial heterogeneity (*I* ^2^ = 94%). The certainty of the evidence was very low (Table S4).

##### 3.5.1.13 HbA1c

**FIGURE 16:**
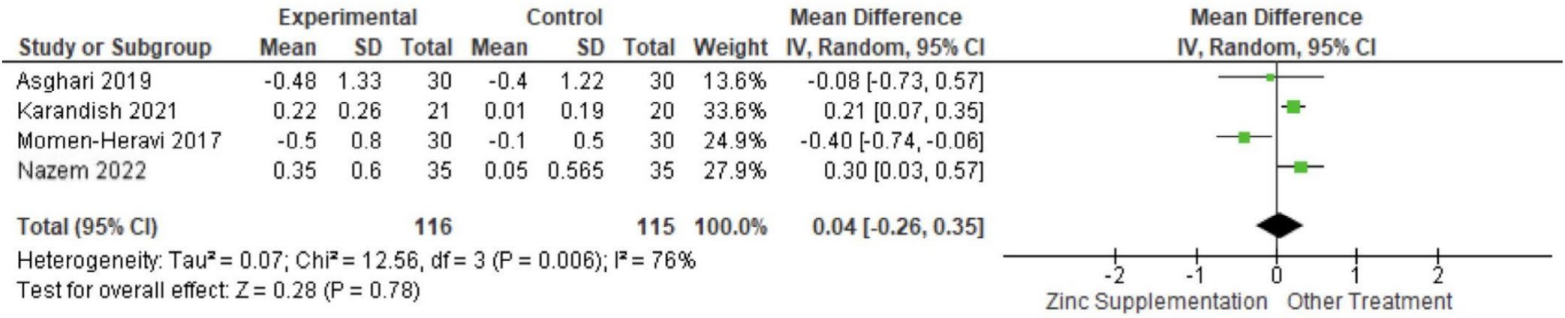
Forest plot hbA1c.

Four studies assessed HbA1c (Figure 16). The available evidence did not demonstrate a significant effect on HbA1c [15, 19, 20, 24] (MD = 0.04; 95% CI −0.26 to 0.35; *p* = 0.78), although interpretation is limited by the small number of studies and substantial heterogeneity. The certainty of the evidence was very low (Table S4).

##### 3.5.1.14 Magnesium

**FIGURE 17:**
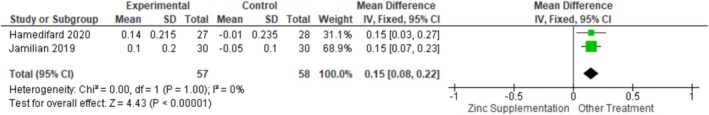
Forest plot magnesium.

Two studies demonstrated significantly higher magnesium levels with zinc supplementation (Figure 17) [12, 16] (MD = 0.15; 95% CI 0.08 to 0.22; *p* < 0.00001), with negligible heterogeneity (*I* ^2^ = 0%). Certainty of evidence was high (Table S4).

##### 3.5.1.15 QUICKI

**FIGURE 18:**
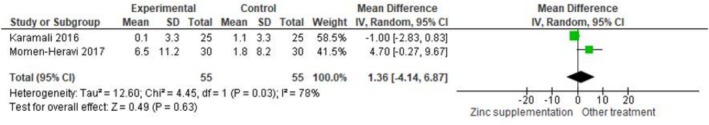
Forest plot QUICKI level.

Two studies assessed QUICKI (Figure 18). No significant differences were observed [17, 19] (MD = 1.36; 95% CI −4.14 to 6.87; *p* = 0.63), with considerable heterogeneity (*I* ^2^ = 78%). The certainty of the evidence was very low (Table S4).

#### 3.5.2 Clinical Parameters

##### 3.5.2.1 Systolic Blood Pressure (SBP)

**FIGURE 19:**
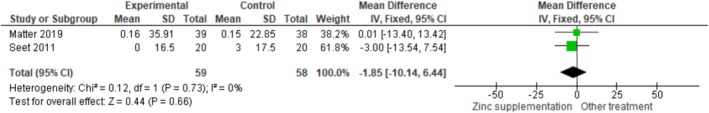
Forest plot SBP (systolic blood pressure).

Two studies assessed SBP (Figure 19). No significant differences were observed [18, 28] (MD = −1.85; 95% CI −10.14 to 6.44; *p* = 0.66; *I* ^2^ = 0%). Certainty of evidence was low (Table S4).

##### 3.5.2.2 Body Mass Index (BMI)

**FIGURE 20:**
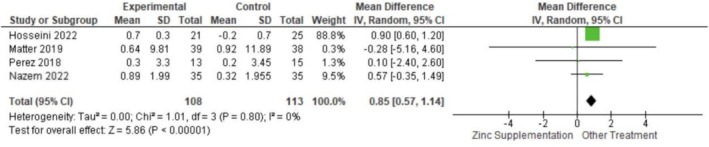
Forest plot BMI.

Four studies reported significantly higher BMI with zinc supplementation (Figure 20) [18, 22, 24, 27] (MD = 0.85; 95% CI 0.57 to 1.14; *p* < 0.00001; *I* ^2^ = 0%). Certainty of evidence was moderate (Table S4). The clinical significance of this increase is unclear in the absence of body composition measures.

##### 3.5.2.3 Body Weight

**FIGURE 21:**
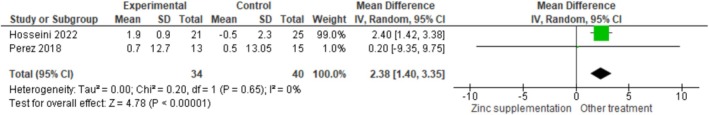
Forest plot weight.

Two studies showed significantly higher body weight with zinc supplementation (Figure 21) [22, 27] (MD = 2.38; 95% CI 1.40 to 3.35; *p* < 0.00001; *I* ^2^ = 0%). Certainty of evidence was low (Table S4).

##### 3.5.2.4 β‐Cell Function

**FIGURE 22:**
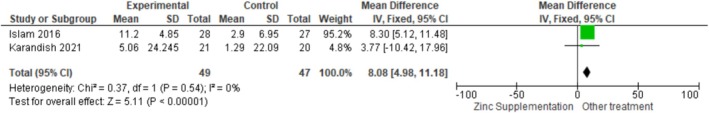
Forest plot beta cell function.

Two studies reported significantly higher β‐cell function with zinc supplementation (Figure 22) [11, 20] (MD = 8.08; 95% CI 4.98 to 11.18; *p* < 0.00001; *I* ^2^ = 0%). Certainty of evidence was moderate (Table S4).

##### 3.5.2.5 Insulin Sensitivity

**FIGURE 23:**
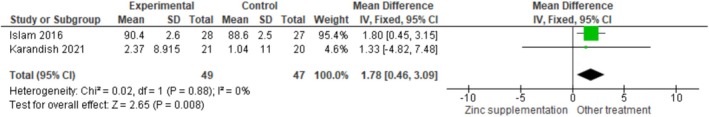
Forest plot insulin sensitivity.

Two studies reported significantly higher insulin sensitivity with zinc supplementation (Figure 23) [11, 20] (MD = 1.78; 95% CI 0.46 to 3.09; *p* = 0.008; *I* ^2^ = 0%). Certainty of evidence was moderate (Table S4).

##### 3.5.2.6 Insulin Resistance

**FIGURE 24:**
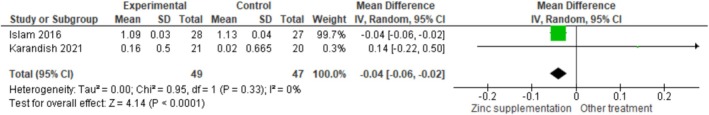
Forest plot insulin resistance.

Two studies reported significantly reduced insulin resistance with zinc supplementation (Figure 24) [11, 20] (MD = −0.04; 95% CI −0.06 to −0.02; *p* < 0.0001; *I* ^2^ = 0%). Certainty of evidence was moderate (Table S4).

#### 3.5.3 Inflammatory and Oxidative Stress Parameters

##### 3.5.3.1 CRP/hs‐CRP

**FIGURE 25:**
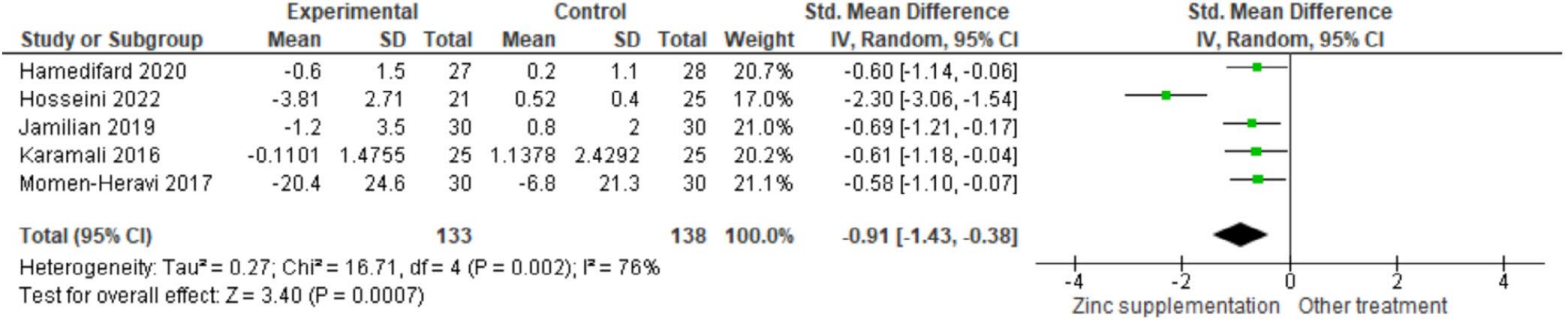
Forest plot CRP/hs‐CRP.

Five studies showed a significant reduction in CRP/hs‐CRP following zinc supplementation (Figure 25) [12, 16, 17, 19, 22] (SMD = −0.91; 95% CI −1.43 to −0.38; *p* = 0.0007), with considerable heterogeneity (*I* ^2^ = 76%). Certainty of evidence was moderate (Table S4).

##### 3.5.3.2 Malondialdehyde (MDA)

**FIGURE 26:**
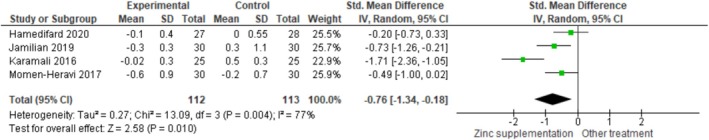
Forest plot MDA/serum MDA (malondialdehyde).

Four studies showed significantly lower MDA with zinc supplementation (Figure 26) [12, 16, 17, 19] (SMD = −0.76; 95% CI −1.34 to −0.18; *p* = 0.010), with substantial heterogeneity (*I* ^2^ = 77%). Certainty of evidence was moderate (Table S4).

##### 3.5.3.3 Nitric Oxide (NO)

**FIGURE 27:**
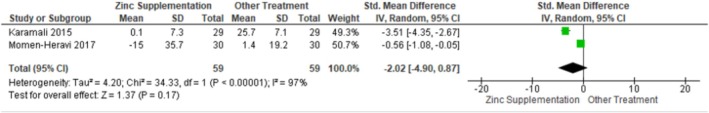
Forest plot NO (nitric oxide) level.

Two studies found no significant effect on nitric oxide (Figure 27) [13, 19] (SMD = −2.02; 95% CI −4.90 to 0.87; *p* = 0.17), with considerable heterogeneity (*I* ^2^ = 97%). Certainty of evidence was low (Table S4).

##### 3.5.3.4 Total Antioxidant Capacity (TAC)

**FIGURE 28:**
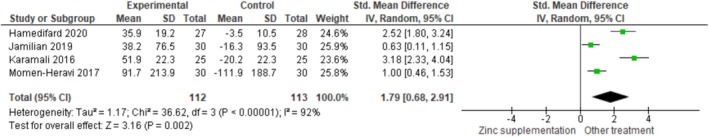
Forest plot TAC (total antioxidant capacity).

Four studies showed significantly higher TAC with zinc supplementation (Figure 28) [12, 16, 17, 19] (SMD = 1.79; 95% CI 0.68 to 2.91; *p* = 0.002), with substantial heterogeneity (*I* ^2^ = 92%). Certainty of evidence was moderate (Table S4).

##### 3.5.3.5 Total Glutathione (GSH)

**FIGURE 29:**
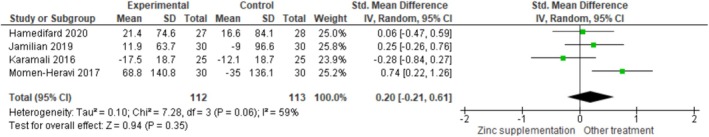
Forest plot GSH (total glutathione).

Four studies showed no significant effect on GSH (Figure 29) [12, 16, 17, 19] (SMD = 0.20; 95% CI −0.21 to 0.61; *p* = 0.35), with moderate heterogeneity (*I* ^2^ = 59%). Certainty of evidence was low (Table S4).

#### 3.5.4 Adverse Effects

Across included trials, zinc supplementation was generally well tolerated. Seven studies explicitly reported no adverse effects during the intervention period. Anderson [14] and Roussel [25] reported no negative impact on copper status or HDL cholesterol [14, 25]. Hamedifard [16], Islam [11], Attia [21], Karandish [20] and Matter [18] similarly reported no significant side effects. The remaining studies did not report safety concerns. Although excessive zinc intake may cause gastrointestinal symptoms and interfere with copper absorption, long‐term safety data in diabetic populations remain limited.

#### 3.5.5 Meta‐Regression

Meta‐regression analyses were conducted to explore sources of heterogeneity using mean age, supplementation duration, daily zinc dose and proportion of women as moderators. Mean age emerged as a significant moderator for multiple lipid, inflammatory and oxidative stress outcomes, including HDL‐cholesterol, LDL‐cholesterol, VLDL‐cholesterol, total cholesterol, triglycerides, hs‐CRP, serum malondialdehyde and HOMA‐IR, suggesting that age may be associated with variability in metabolic and redox responses to zinc supplementation. Sex distribution also significantly moderated several outcomes. A higher proportion of women was associated with lower circulating zinc concentrations, reduced total glutathione, decreased insulin sensitivity (QUICKI), and higher HbA1c values, indicating potential sex‐specific differences in zinc metabolism and glycemic response. Supplementation duration significantly moderated LDL‐cholesterol only, with longer interventions associated with modest reductions. Daily zinc dose was not a significant predictor of most biochemical or metabolic outcomes, except for BMI, where higher doses were associated with small increases in body mass index. Overall, age and sex distribution accounted for more between‐study variability than zinc dose or intervention duration, underscoring the importance of demographic factors when interpreting the observed metabolic associations of zinc supplementation in diabetic populations (Table 2 and Table S5).

**TABLE 2:** Significant moderators of zinc supplementation effects (meta‐regression).

Outcome	Moderator	*B*	95% CI	*p*
Zinc concentration	Proportion of women	−9.03	−17.82 to −0.23	0.044
HDL‐cholesterol	Mean age (years)	0.00	0.00 to 0.00	0.013
LDL‐cholesterol	Mean age (years)	0.00	0.00 to 0.00	0.012
LDL‐cholesterol	Supplementation duration (weeks)	−0.02	−0.04 to 0.00	0.045
VLDL‐cholesterol	Mean age (years)	0.00	0.00 to 0.00	< 0.001
Total cholesterol	Mean age (years)	0.00	0.00 to 0.00	< 0.001
Triglycerides	Mean age (years)	−0.02	−0.03 to −0.01	0.004
QUICKI	Proportion of women	−0.01	−0.03 to 0.00	0.023
GSH	Proportion of women	−1.02	−1.88 to −0.16	0.020
BMI	Daily zinc dose (mg/day)	0.01	0.00 to 0.01	< 0.001
hs‐CRP	Mean age (years)	0.00	0.00 to 0.00	0.006
Serum MDA	Mean age (years)	0.00	0.00 to 0.00	0.001
HOMA‐IR	Mean age (years)	0.08	0.00 to 0.16	0.042
HbA1c	Proportion of women	4.36	2.10 to 6.61	< 0.001

*Note:* Only moderators with *p* < 0.05 are shown. Regression coefficient (*B*) represents the change in the pooled effect estimate per unit increase in the moderator. Full meta‐regression results are presented in Table S5. Only moderators with *p* < 0.05 are shown. Full meta‐regression results are presented in Table S5.

## 4 Discussion

### 4.1 Principal Findings

This systematic review and meta‐analysis evaluated the effects of zinc supplementation on metabolic, inflammatory, and oxidative stress outcomes in individuals with diabetes, gestational diabetes or prediabetes. Overall, zinc supplementation was associated with improvements in markers of insulin resistance and inflammatory/oxidative stress. Specifically, pooled analyses showed reductions in serum insulin (MD = −2.50 μU/mL; 95% CI −4.69 to −0.31) and HOMA‐IR (MD = −1.10; 95% CI −2.05 to −0.15), accompanied by lower CRP/hs‐CRP (SMD = −0.91; 95% CI −1.43 to −0.38) and malondialdehyde (SMD = −0.76; 95% CI −1.34 to −0.18). Total antioxidant capacity increased (SMD = 1.79; 95% CI 0.68 to 2.91), supporting a potential antioxidant effect.

In contrast, fasting plasma glucose and HbA1c did not demonstrate significant improvement (FPG: MD = −4.35 mg/dL; 95% CI −10.80 to 2.11; HbA1c: MD = 0.04%; 95% CI −0.26 to 0.35), and lipid outcomes were mixed. Total cholesterol decreased (MD = −5.70 mg/dL; 95% CI −7.50 to −3.89), whereas LDL cholesterol showed a modest increase (MD = 3.46 mg/dL; 95% CI 1.48 to 5.43). Although statistically significant, the absolute reduction in total cholesterol was relatively modest and may have limited clinical impact at the individual patient level. The clinical relevance of the observed LDL increase also remains uncertain. Although the absolute increase was relatively small, LDL cholesterol is a recognized cardiovascular risk marker, and its elevation may partially offset potential metabolic or anti‐inflammatory benefits associated with zinc supplementation. Because apoB, LDL particle size, oxidized LDL, and cardiovascular outcomes were not consistently reported, the long‐term vascular implications of this finding cannot be determined (Table 3).

**TABLE 3:** Variables and parameters for diabetes mellitus.

Study variables		Status of the parameter in diabetic individuals	Favourable outcome
Biochemical parameters	Serum Magnesium (mg/dL or mmol/L)	Low or at the lower limit	Normalized or Slightly Elevated (1.7–2.3 mg/dL or 0.7–0.95 mmol/L)
	Serum calcium (mmol/L)	Slightly low (related to hypomagnesemia)	Normal or improved with the electrolyte balance (2.1–2.6 mmol/L)
	Serum zinc (μg/dL)	Low, associated with inflammation and oxidative stress	Increase towards normal values (70–120 μg/dL)
	FPG (mg/dL)	High (> 126 mg/dL)	Reduction towards controlled values (< 126 mg/dL)
	HbA1c (%)	High (> 6.5%)	Reduction towards therapeutic targets (< 7%)
	Triglycerides (mg/dL)	High (> 150 mg/dL)	Decrease towards normal values (< 150 mg/dL)
	Total cholesterol (mg/dL)	High (> 200 mg/dL)	Reduction to desirable values (< 200 mg/dL)
	VLDL‐cholesterol (mg/dL)	High (> 30 mg/dL, associated with hypertriglyceridemia)	Decrease to normal ranges (< 30 mg/dL)
	LDL‐cholesterol (mg/dL)	High (> 100–130 mg/dL depending on risk)	Reduction towards therapeutic targets (< 100 mg/dL)
	HDL‐cholesterol (mg/dL)	Low (< 40 mg/dL in men, < 50 mg/dL in women)	Increase (> 40 mg/dL in men, > 50 mg/dL in women)
	Insulin (μIU/mL)	High (indicating insulin resistance)	Decrease to normal ranges (< 25 μIU/mL)
Clinical indicators	BMI (kg/m^2^)	Frequent overweight/obesity	Possible reduction with better metabolic control
	SBP/DBP (mmHg)	High (> 130/80 mmHg)	Reduction to normal values (< 130/80 mmHg)
	Ulcer width and depth (cm)	Wider and deeper (slower healing)	Faster healing, with reduced size
	ESR (mm/h)	High (> 20 mm/h in women, > 15 mm/h in men)	Reduction to normal values (< 20 mm/h)
Inflammation and oxidative stress markers	hs‐CRP (mg/L)	High (> 3 mg/L, indicating chronic inflammation)	Reduction (< 1 mg/L, indicating lower inflammation)
	Nitric oxide (μmol/L)	Low (indicating endothelial dysfunction)	Increase (better vascular function)
	TAC (mmol/L)	Low (indicating lower antioxidant capacity)	Increase (indicating higher antioxidant capacity)
	GSH (μmol/L)	Low (indicating depletion of antioxidants)	Increase (indicating better antioxidant protection)
	MDA (μmol/L)	High (indicating higher oxidative stress)	Decrease (indicating lower oxidative damage)
Calculated ratios	QUICKI	Low (indicating insulin resistance)	Increase (better insulin sensitivity)
	HOMA‐IR	High (indicating insulin resistance)	Decrease (< 2.5 in non‐obese individuals)
	Total/HDL cholesterol ratio	High (> 4.5, indicating increased cardiovascular risk)	Decrease (< 3.5, indicating lower risk)

Abbreviations: BMI, Body mass index; DBP, Diastolic Blood Pressure; ESR, Erythrocyte sedimentation rate (mm/h); FPG, Fasting plasma glucose; GSH, total glutathione; HbA1c, Glycosylated haemoglobin; HOMA‐IR, Homeostasis Model Assessment–Insulin Resistance; hs‐CRP, C‐reactive protein; MDA, malondialdehyde; QUICKI, quantitative insulin sensitivity check index; SBP, Systolic Blood Pressure; T2DM, Type 2 Diabetes Mellitus; TAC, total antioxidant capacity.

From an evidence certainty perspective (GRADE), reductions in insulin, HOMA‐IR, CRP/hs‐CRP, TAC and MDA were supported by moderate‐certainty evidence, while effects on fasting glucose, HbA1c and QUICKI were supported by low‐to‐very‐low certainty evidence, primarily due to inconsistency and imprecision. Collectively, the findings suggest that zinc supplementation may modulate insulin resistance and inflammatory/oxidative pathways in diabetic populations, although effects on glycemic control and lipid subclasses remain variable. Adequately powered, longer‐duration trials are required before broad clinical recommendations can be supported.

Importantly, interpretation of these pooled effects must remain cautious because several outcomes were supported by low or very low certainty evidence, and many included studies presented limitations in allocation concealment, blinding and overall methodological rigour. Moreover, interpretation of pooled effect sizes is complicated by the substantial heterogeneity observed across several outcomes, including plasma zinc concentrations, insulin, HOMA‐IR, CRP/hs‐CRP, MDA and TAC. The wide variability in effect estimates across studies suggests that individual responses to zinc supplementation may differ considerably according to diabetes subtype, baseline metabolic status, nutritional profile, co‐interventions and intervention duration.

### 4.2 Comparison With Previous Evidence

The present results are broadly consistent with previous evidence indicating that zinc supplementation may improve insulin sensitivity and redox balance, although effect sizes vary across populations and study designs. Prior meta‐analyses have reported favourable effects of zinc on insulin resistance indices and selected glycemic outcomes in type 2 diabetes and prediabetes, including reductions in fasting plasma glucose, HOMA‐IR and insulin in some datasets [2]. Other syntheses have suggested modest glycemic benefits (including HbA1c reductions) under specific conditions such as longer duration, baseline zinc deficiency or more homogeneous populations [3]. Our analysis extends this literature by incorporating a broader range of outcomes, particularly inflammatory and oxidative stress markers, and demonstrates consistent directionality towards lower CRP/hs‐CRP and MDA and higher TAC, despite substantial heterogeneity for several endpoints. In gestational diabetes, prior reports of improvements in oxidative stress markers align with our observed reduction in malondialdehyde [4]. Differences across meta‐analyses likely reflect variation in diabetes phenotype, zinc formulation, and elemental dose, intervention duration, baseline nutritional status, and whether zinc was administered as a standalone supplement or within co‐supplement regimens.

Co‐supplementation (e.g., magnesium, vitamin D, curcumin or vitamin A) may plausibly amplify or obscure zinc‐specific effects, thereby limiting attribution of observed metabolic changes to zinc alone. Recent umbrella reviews and meta‐analyses have demonstrated that several nutraceutical interventions, including curcumin, *Berberis* species and *Nigella sativa* , may independently improve inflammatory biomarkers, oxidative stress parameters, insulin resistance and selected glycemic outcomes in metabolic disease populations [32, 33, 34, 35]. For example, curcumin supplementation has been associated with reductions in CRP, IL‐6, TNF‐α and MDA, alongside improvements in antioxidant enzyme activity and endothelial function [32, 33], whereas *Berberis* and *Nigella sativa* supplementation have demonstrated potential benefits across glycemic indices, lipid parameters, body weight and insulin resistance markers in patients with type 2 diabetes and related metabolic disorders [34, 35]. Consequently, the magnitude of zinc‐specific effects in the present analysis may have been partially overestimated in studies using multicomponent supplementation protocols.

Baseline zinc status likely represents an important effect modifier that may partially explain the variability observed across studies. Individuals with zinc deficiency may experience greater metabolic and antioxidant benefits from supplementation than zinc‐replete populations, in whom physiological responses could be attenuated. Therefore, pooling participants regardless of baseline zinc status may have diluted potentially clinically relevant effects in deficient subgroups.

### 4.3 Biological Plausibility

Zinc has well‐established roles in glucose metabolism, insulin action and oxidative defence, which support the biological plausibility of the observed associations. Zinc contributes to pancreatic β‐cell physiology by supporting insulin synthesis, crystallization and storage, and may protect β‐cells from apoptosis under oxidative stress [30]. In peripheral tissues, zinc participates in insulin signalling pathways, including PI3K/Akt‐related mechanisms, improving insulin sensitivity [29]. These pathways align with reductions in serum insulin and HOMA‐IR, suggesting zinc may reduce compensatory hyperinsulinemia by enhancing insulin action rather than stimulating insulin secretion. Zinc also supports antioxidant systems through its structural and regulatory roles in enzymes involved in reactive oxygen species detoxification, such as superoxide dismutase and catalase [31]. The observed increase in TAC and reduction in MDA are consistent with reduced lipid peroxidation and oxidative injury. Because oxidative stress and chronic low‐grade inflammation contribute to diabetic complications, zinc's redox and inflammatory effects may be clinically meaningful even when short‐term HbA1c changes are not observed.

### 4.4 Interpretation of Discordant Findings

Several outcomes observed in this meta‐analysis warrant careful physiological interpretation, particularly where apparently discordant metabolic responses were identified. First, the absence of consistent significant improvement in fasting glucose and HbA1c despite reductions in insulin and HOMA‐IR likely reflects the complex temporal dynamics of glycemic regulation. Improvements in insulin sensitivity may precede measurable changes in glycemic indices, particularly HbA1c, which reflects cumulative glucose exposure over approximately 3 months and may require longer intervention periods to demonstrate sustained improvement. Furthermore, HOMA‐IR primarily represents surrogate estimates of insulin resistance derived from fasting insulin and glucose concentrations and may therefore respond earlier to metabolic modulation than downstream glycemic endpoints. Variability in baseline glycemic control, diabetes phenotype, intervention duration and concomitant therapies across trials likely further contributed to these discordant findings.

Second, lipid responses showed a seemingly paradoxical pattern, characterized by reductions in total cholesterol alongside modest increases in LDL cholesterol. In the absence of detailed lipoprotein profiling, including apolipoprotein B, LDL particle number or size, non‐HDL cholesterol and oxidized LDL, these findings cannot be reliably interpreted in terms of cardiovascular risk. It is plausible that changes in LDL cholesterol reflect shifts in particle composition rather than atherogenic burden; however, without consistent reporting of these parameters, the clinical relevance of this pattern remains uncertain. Although the absolute increase in LDL cholesterol was relatively small, LDL cholesterol remains a recognized cardiovascular risk marker, and its elevation may partially offset potential metabolic or anti‐inflammatory benefits associated with zinc supplementation. Future trials should incorporate comprehensive lipoprotein metrics to enable more precise cardiovascular risk stratification. Taken together, these discordant findings complicate the overall clinical interpretation of zinc supplementation in diabetes. Although improvements in insulin resistance, inflammation and oxidative stress may suggest potential metabolic benefit, the concomitant increases in LDL cholesterol, body weight and BMI raise uncertainty regarding the net cardiometabolic effect. In the absence of data on cardiovascular outcomes, body composition or long‐term vascular risk, the clinical balance between potential benefits and harms remains unclear.

Third, pooled analyses indicated small but statistically significant increases in body weight and BMI following zinc supplementation. Without concurrent assessment of body composition, dietary intake, energy balance or baseline zinc deficiency, these changes cannot be classified as either beneficial or adverse. In zinc‐deficient individuals, modest weight gain may reflect improved nutritional status or anabolic recovery, whereas in zinc‐replete populations it could represent undesirable increases in adiposity. The absence of standardized anthropometric and nutritional assessments limits the interpretation of these findings. Therefore, these anthropometric findings should not be interpreted as inherently beneficial, particularly in populations already at elevated cardiometabolic risk.

Finally, the observed reduction in HOMA‐B, concurrent with decreases in insulin and HOMA‐IR, underscores the dual physiological interpretation of β‐cell function indices. Because HOMA‐B reflects both β‐cell secretory capacity and compensatory insulin demand, a reduction may indicate decreased β‐cell workload secondary to improved insulin sensitivity rather than impaired β‐cell function. However, given the limited number of contributing studies and the low certainty of evidence for this outcome, conclusions regarding direct effects of zinc supplementation on β‐cell function should remain conservative. Taken together, the current evidence supports a potentially favourable but uncertain role for zinc supplementation in modulating selected metabolic and inflammatory pathways in diabetes‐related conditions. However, substantial heterogeneity, methodological limitations, co‐intervention effects and variability in evidence certainty preclude definitive conclusions regarding overall clinical efficacy.

## 5 Limitations

Notwithstanding the physiological considerations discussed above, several methodological limitations should be acknowledged when interpreting the findings of this meta‐analysis. First, there was substantial clinical and methodological heterogeneity across the included trials, encompassing differences in diabetes phenotype (type 1 diabetes, type 2 diabetes, gestational diabetes and prediabetes), participant characteristics, zinc formulation and elemental dose, intervention duration and the frequent use of zinc in combination with other micronutrients or bioactive compounds. This heterogeneity likely contributed to the considerable inconsistency observed for multiple outcomes and limits the generalizability of pooled effect estimates.

An additional limitation relates to the inclusion of multicomponent supplementation trials. Several studies administered zinc alongside other micronutrients or bioactive compounds, including magnesium, vitamin D, calcium, curcumin and vitamin A. Because these agents may independently affect glycemic control, inflammation, oxidative stress and lipid metabolism, the isolated contribution of zinc could not be definitively determined.

Second, many meta‐analyses were based on a relatively small number of studies and participants, resulting in wide confidence intervals and imprecision of effect estimates. Consequently, several outcomes were downgraded within the GRADE framework, reflecting uncertainty in the magnitude and robustness of the observed effects. This limitation is particularly relevant for secondary outcomes and subgroup analyses, which should be interpreted as exploratory rather than confirmatory. Additionally, the relatively small number of studies and participants available for glycemic outcomes such as fasting glucose and HbA1c limited statistical power and may have increased the risk of type II error, precluding exclusion of small but potentially clinically relevant effects.

Third, outcome measurement and reporting were not uniform across trials. Variability in laboratory assays, diagnostic thresholds, and reporting formats necessitated the transformation or imputation of missing statistical parameters in some analyses, introducing additional uncertainty. Although standard methodological approaches were applied, these procedures may have contributed to residual imprecision and heterogeneity, particularly for inflammatory and oxidative stress biomarkers.

Fourth, baseline zinc status was inconsistently assessed and reported across studies, precluding stratified analyses according to zinc deficiency or sufficiency. Because physiological and metabolic responses to supplementation are likely to differ between zinc‐deficient and zinc‐replete individuals, failure to account for baseline zinc status may have diluted potentially clinically relevant effects in deficient populations and contributed to the substantial heterogeneity observed across outcomes. This limitation restricts inference regarding which patient subgroups may derive the greatest benefit from zinc supplementation [36].

Fifth, the duration of most interventions was short to moderate, which restricts the evaluation of long‐term metabolic, glycemic and vascular outcomes. While improvements in insulin resistance, inflammatory markers and oxidative stress may represent early or intermediate biological effects [37], longer follow‐up periods are required to determine whether these changes translate into sustained improvements in glycemic control, cardiovascular risk or clinical endpoints [38, 39].

Finally, although comprehensive searches were conducted across multiple databases, the possibility of publication bias cannot be excluded. Formal assessment of small‐study effects was limited by the relatively small number of studies available for several outcomes, and language restrictions may have resulted in the exclusion of potentially relevant evidence. In addition, several pooled analyses demonstrated considerable statistical heterogeneity despite subgroup and meta‐regression analyses. This residual heterogeneity suggests that important sources of variability may remain unidentified and reduces confidence in the consistency, predictability and generalizability of pooled treatment effects across different clinical populations. These methodological considerations should be taken into account when interpreting the overall strength and applicability of the present findings.

## 6 Future Directions

Future research on zinc supplementation in diabetes should prioritize methodological rigour and clinical relevance through more targeted trial designs. Multicenter randomized controlled trials with adequate sample sizes are needed to enhance external validity and reduce imprecision, particularly for secondary metabolic, inflammatory and oxidative stress outcomes. Harmonized eligibility criteria would facilitate comparison across studies and improve the interpretability of pooled evidence.

Intervention protocols should be prespecified with greater precision, including justification of elemental zinc dose, chemical form, dosing schedule and duration based on pharmacokinetic and physiological considerations. Consistent monitoring of adherence, adverse events and potential interactions with background dietary intake or antidiabetic medications is essential to contextualize efficacy and safety.

From an outcome's perspective, future trials should adopt predefined core outcome frameworks aligned with diabetes research priorities. In addition to standard glycemic markers, the inclusion of validated surrogate endpoints related to vascular health, endothelial function and microvascular damage would help bridge the gap between short‐term biochemical effects and clinically meaningful benefits. Reporting of outcomes should follow standardized templates to minimize selective reporting and facilitate synthesis.

Given the frequent use of combined micronutrient interventions in existing studies, future trials should explicitly distinguish between zinc‐only and multicomponent strategies at the design stage. Factorial or multi‐arm randomized designs would allow formal testing of interaction effects and enable clearer attribution of observed benefits or harms. In addition, the number of studies per outcome was insufficient to reliably assess publication bias. Finally, integration of mechanistic and translational endpoints, such as zinc transporter expression, markers of cellular oxidative balance and inflammatory signalling pathways, may help clarify biological responsiveness across patient subgroups. Such approaches could support precision nutrition strategies and help determine whether zinc supplementation is better positioned as a targeted intervention for specific diabetic populations rather than as a universal adjunctive therapy. Future studies should prioritize more homogeneous patient populations and standardized intervention protocols to reduce between‐study variability and improve the interpretability of pooled estimates. In addition, future trials should systematically assess and report baseline zinc status to determine whether supplementation benefits differ between zinc‐deficient and zinc‐sufficient individuals.

## 7 Conclusions

Zinc supplementation was associated with improvements in insulin resistance, inflammatory status and oxidative stress markers in individuals with diabetes, gestational diabetes or prediabetes, including reductions in serum insulin, HOMA‐IR, CRP/hs‐CRP and malondialdehyde, alongside increases in total antioxidant capacity. However, fasting glucose and HbA1c did not demonstrate consistent significant improvement, and LDL cholesterol increased modestly, indicating that potential benefits may not extend uniformly across glycemic and lipid‐related outcomes. Furthermore, the inclusion of multicomponent supplementation studies limits the ability to isolate zinc‐specific effects.

Given the substantial heterogeneity across studies, frequent methodological limitations and predominantly low‐to‐moderate certainty of evidence for most clinically relevant outcomes, these findings should be interpreted cautiously. Additionally, the observed increases in LDL cholesterol, BMI and body weight warrant further investigation before definitive conclusions regarding the overall cardiometabolic impact of zinc supplementation can be established. Although zinc supplementation may represent a potential adjunctive nutritional strategy for modulating selected metabolic and inflammatory pathways, current evidence remains insufficient to support routine clinical recommendations until larger, rigorously designed randomized controlled trials with longer follow‐up are available.

## Author Contributions

**Matias Donoso‐Emig:** conceptualization, investigation, writing – review and editing. **Jessica Paola Loaiza‐Giraldo:** conceptualization, investigation, methodology, validation, formal analysis, supervision. **Pablo Nova‐Baeza:** conceptualization, investigation, validation, data curation. **Alejandro Bruna‐Mejias:** conceptualization, validation, formal analysis, data curation. **Mathias Orellana‐Donoso:** data curation, conceptualization, methodology, investigation. **Maria Piagkou:** conceptualization, validation, methodology, software, formal analysis. **Camila Ignacia Cancino‐Castro:** conceptualization, methodology, software, project administration, writing – review and editing. **Ignacia Farías‐Quinteros:** conceptualization, methodology, software. **Juan Sanchis‐Gimeno:** conceptualization, validation, formal analysis, supervision. **Vitor E. Valenti:** conceptualization, investigation, funding acquisition, writing – original draft, validation. **Jose E. Leon‐Rojas:** methodology, writing – review and editing, writing – original draft, resources. **Juan José Valenzuela‐Fuenzalida:** conceptualization, methodology, resources, supervision, formal analysis. **Florencia Amigo‐Fierro:** conceptualization, methodology, software, project administration. **Héctor Gutiérrez‐Espinoza:** conceptualization, investigation, formal analysis, visualization. **Murtaja Satea:** conceptualization, writing – original draft, writing – review and editing. **Gloria Cifuentes‐Suazo:** conceptualization, methodology, formal analysis, supervision.

## Funding

The authors have nothing to report.

## Conflicts of Interest

The authors declare no conflicts of interest.

## Supporting information


**Table S1:** Search strategy for identification of eligible studies.
**Table S2:** Excluded studies and the reasons for their exclusion.
**Table S3:** Detailed characteristics of included randomized controlled trials.
**Table S4:** Summary of Findings (SoF) and GRADE certainty of evidence for zinc supplementation in diabetic individuals.
**Table S5:** Complete moderator analyses.

## Data Availability

The data that support the findings of this study are available from the corresponding author upon reasonable request.
