# Supplementary material for: Effects of Zinc Supplementation on Glycemic Control, Insulin Resistance, Inflammation and Oxidative Stress in Diabetes: A Systematic Review and Meta‐Analysis of Randomized Controlled Trials
Source: Endocrinol Diabetes Metab. 2026 Aug 13;9(5):e70264. doi: 10.1002/edm2.70264 (PMC13472645; doi:10.1002/edm2.70264)
Supplement: Supplementary file 1 [file EDM2-9-e70264-s001.docx]

**Table S1: Search strategy for identification of eligible studies**

| **Database** | **Search strategy** | **Results** | |
| --- | --- | --- | --- |
|  |  | **20-10-24** | **19-01-2024** |
| Medline | zinc supplementation AND diabetes Filters: Clinical Trial, Randomized Controlled Trial, English, Humans  (("zinc"[Supplementary Concept] OR "zinc"[All Fields] OR "zinc"[MeSH Terms]) AND ("supplemental"[All Fields] OR "supplementating"[All Fields] OR "supplementation"[All Fields] OR "supplementation s"[All Fields] OR "supplementations"[All Fields] OR "supplemention"[All Fields]) AND ("diabete"[All Fields] OR "diabetes mellitus"[MeSH Terms] OR ("diabetes"[All Fields] AND "mellitus"[All Fields]) OR "diabetes mellitus"[All Fields] OR "diabetes"[All Fields] OR "diabetes insipidus"[MeSH Terms] OR ("diabetes"[All Fields] AND "insipidus"[All Fields]) OR "diabetes insipidus"[All Fields] OR "diabetic"[All Fields] OR "diabetics"[All Fields] OR "diabets"[All Fields])) AND ((clinicaltrial[Filter] OR randomizedcontrolledtrial[Filter]) AND (humans[Filter]) AND (english[Filter])) | 70 | 70 |
| Wos | zinc supplementation AND diabetes Filters: Clinical Trial, Randomized Controlled Trial, English, Humans | 33 | 33 |
| CINAHL | zinc supplementation AND diabetes Filters: Clinical Trial, Randomized Controlled Trial, English, Humans | 12 | 12 |

| SCOPUS | zinc supplementation AND diabetes Filters: Clinical Trial, Randomized Controlled Trial, English, Humans | 95 | 96 |
| --- | --- | --- | --- |
| Google Scholar | zinc supplementation AND diabetes Filters: Clinical Trial, Randomized Controlled Trial, English, Humans | 88 | 89 |
|  | Total | 298 | 300 |

All searches were carried out on January 20, 2026.

**Table S2:** Excluded studies and the reasons for their exclusion.

|  | **Reference** | **Reason** |
| --- | --- | --- |
| 1 | Kim HN, Kim SH, Eun YM, Song SW. Effects of zinc, magnesium, and chromium supplementation on cardiometabolic risk in adults with metabolic syndrome: A double-blind, placebo-controlled randomised trial. J Trace Elem Med Biol. 2018 Jul;48:166-171. doi: 10.1016/j.jtemb.2018.03.022. Epub 2018 Mar 30. PMID: 29773176. | Patients with metabolic syndrome but not with diabetes |
| 2 | Sivakumar RR, Chinnaiah Govindareddy D, Sahoo J, Bobby Z, Chinnakali P. Effect of daily zinc supplementation for 12 weeks on serum thyroid auto-antibody levels in children and adolescents with autoimmune thyroiditis - a randomized controlled trial. J Pediatr Endocrinol Metab. 2024 Jan 1;37(2):137-143. doi: 10.1515/jpem-2023-0165. PMID: 38154030. | **Not a diabetes or prediabetes population (autoimmune thyroiditis)** |
| 3 | Karamali M, Bahramimoghadam S, Sharifzadeh F, Asemi Z. Magnesium-zinc-calcium-vitamin D co-supplementation improves glycemic control and markers of cardiometabolic risk in gestational diabetes: a randomized, double-blind, placebo-controlled trial. Appl Physiol Nutr Metab. 2018 Jun;43(6):565-570. doi: 10.1139/apnm-2017-0521. Epub 2018 Jan 9. Retraction in: Appl Physiol Nutr Metab. 2025 Jan 1;50:1. doi: 10.1139/apnm-2025-0087. PMID: 29316405. | Retraction article |
| 4 | Heidarzadeh Z, Samimi M, Seifati SM, Ashkezari MD, Ahmadi S, Mahmoodi S, Aghadavod E, Jamilian M, Asemi Z. The Effect of Zinc Supplementation on Expressed Levels of Peroxisome Proliferator-Activated Receptor Gamma and Glucose Transporter Type 1 Genes in Newborns of Women with Gestational Diabetes Mellitus. Biol Trace Elem Res. 2017 Feb;175(2):271-277. doi: 10.1007/s12011-016-0788-y. Epub 2016 Jun 22. Retraction in: Biol Trace Elem Res. 2023 Sep;201(9):4623-4624. doi: 10.1007/s12011-022-03501-2. PMID: 27334434. | **Neonatal population, not diabetes intervention (retracted)** |
| 5 | Basu A, Betts NM, Mulugeta A, Tong C, Newman E, Lyons TJ. Green tea supplementation increases glutathione and plasma antioxidant capacity in adults with the metabolic syndrome. Nutr Res. 2013 Mar;33(3):180-7. doi: 10.1016/j.nutres.2012.12.010. Epub 2013 Jan 30. PMID: 23507223; PMCID: PMC3603270. | Patients with metabolic syndrome but not with diabetes. |
| 6 | Foroozanfard F, Jamilian M, Jafari Z, Khassaf A, Hosseini A, Khorammian H, Asemi Z. Effects of zinc supplementation on markers of insulin resistance and lipid profiles in women with polycystic ovary syndrome: a randomized, double-blind, placebo-controlled trial. Exp Clin Endocrinol Diabetes. 2015 Apr;123(4):215-220. doi: 10.1055/s-0035-1548790. Epub 2015 Apr 13. PMID: 25868059. | Patients with metabolic syndrome but not with diabetes. |
| 7 | Maktabi M, Jamilian M, Asemi Z. Magnesium-Zinc-Calcium-Vitamin D Co-supplementation Improves Hormonal Profiles, Biomarkers of Inflammation and Oxidative Stress in Women with Polycystic Ovary Syndrome: a Randomized, Double-Blind, Placebo-Controlled Trial. Biol Trace Elem Res. 2018 Mar;182(1):21-28. doi: 10.1007/s12011-017-1085-0. Epub 2017 Jul 1. Retraction in: Biol Trace Elem Res. 2023 Aug;201(8):4202-4203. doi: 10.1007/s12011-022-03465-3. Erratum in: Integr Psychol Behav Sci. 2024 Dec;58(4):2057-2058. doi: 10.1007/s12124-022-09733-0. PMID: 28668998. | Retraction article |
| 8 | Czernichow S, Vergnaud AC, Galan P, Arnaud J, Favier A, Faure H, Huxley R, Hercberg S, Ahluwalia N. Effects of long-term antioxidant supplementation and association of serum antioxidant concentrations with risk of metabolic syndrome in adults. Am J Clin Nutr. 2009 Aug;90(2):329-35. doi: 10.3945/ajcn.2009.27635. Epub 2009 Jun 2. PMID: 19491388. | Patients with metabolic syndrome but not with diabetes. |
| 9 | Afshar Ebrahimi F, Foroozanfard F, Aghadavod E, Bahmani F, Asemi Z. The Effects of Magnesium and Zinc Co-Supplementation on Biomarkers of Inflammation and Oxidative Stress, and Gene Expression Related to Inflammation in Polycystic Ovary Syndrome: a Randomized Controlled Clinical Trial. Biol Trace Elem Res. 2018 Aug;184(2):300-307. doi: 10.1007/s12011-017-1198-5. Epub 2017 Nov 10. PMID: 29127547. | Patients with metabolic syndrome but not with diabetes. |
| 10 | Meksawan K, Sermsri U, Chanvorachote P. Zinc supplementation improves anticancer activity of monocytes in type-2 diabetic patients with metabolic syndrome. Anticancer Res. 2014 Jan;34(1):295-9. PMID: 24403477. | Patients with metabolic syndrome but not with diabetes. |
| 11 | Kelishadi R, Hashemipour M, Adeli K, Tavakoli N, Movahedian-Attar A, Shapouri J, Poursafa P, Rouzbahani A. Effect of zinc supplementation on markers of insulin resistance, oxidative stress, and inflammation among prepubescent children with metabolic syndrome. Metab Syndr Relat Disord. 2010 Dec;8(6):505-10. doi: 10.1089/met.2010.0020. Epub 2010 Oct 28. PMID: 21028969. | Patients with metabolic syndrome but not with diabetes. |
| 12 | Jamilian M, Maktabi M, Asemi Z. A Trial on The Effects of Magnesium-Zinc-Calcium-Vitamin D Co-Supplementation on Glycemic Control and Markers of Cardio-Metabolic Risk in Women with Polycystic Ovary Syndrome. Arch Iran Med. 2017 Oct;20(10):640-645. PMID: 29137465. | Patients with metabolic syndrome but not with diabetes. |
| 13 | Hashemipour M, Kelishadi R, Shapouri J, Sarrafzadegan N, Amini M, Tavakoli N, Movahedian-Attar A, Mirmoghtadaee P, Poursafa P. Effect of zinc supplementation on insulin resistance and components of the metabolic syndrome in prepubertal obese children. Hormones (Athens). 2009 Oct-Dec;8(4):279-85. doi: 10.14310/horm.2002.1244. PMID: 20045801. | Patients with metabolic syndrome but not with diabetes. |
| 14 | Fathi M, Alavinejad P, Haidari Z, Amani R. The effects of zinc supplementation on metabolic profile and oxidative stress in overweight/obese patients with non-alcoholic fatty liver disease: A randomized, double-blind, placebo-controlled trial. J Trace Elem Med Biol. 2020 Dec;62:126635. doi: 10.1016/j.jtemb.2020.126635. Epub 2020 Aug 28. PMID: 32932174.. | Patients with metabolic syndrome but not with diabetes. |
| 15 | Fogacci F, Giovannini M, Di Micoli V, Grandi E, Borghi C, Cicero AFG. Effect of Supplementation of a Butyrate-Based Formula in Individuals with Liver Steatosis and Metabolic Syndrome: A Randomized Double-Blind Placebo-Controlled Clinical Trial. Nutrients. 2024 Jul 28;16(15):2454. doi: 10.3390/nu16152454. PMID: 39125336; PMCID: PMC11313833. | Patients with metabolic syndrome but not with diabetes. |

**Table S3:** Detailed characteristics of included randomized controlled trials.

| **Author, year** | **Country** | **Population** | | **Intervention** | | **Outcomes** | **Follow -up** | **Results** |
| --- | --- | --- | --- | --- | --- | --- | --- | --- |
|  |  | **Sample size (n)** | **Patients**  **mean age (SD)** | **Type of intervention** | **Characteristics and doses** |  |  |  |
| Karamali et al., 2015 | Iran | CG: 29  EG:29 | CG: 29.4 ± 3.6  EG: 29.9 ± 5.2  Women with GDM | CG: Placebo.  EG: Supplementation of zinc | CG: Placebo daily for 6 weeks.  EG: 233 mg zinc gluconate (containing 30 mg zinc) daily for 6 weeks. | Zinc  FPG  Insulin  HOMA-IR  HOMA-B  QUICKI  Triglycerides  VLDL-cholesterol  Total cholesterol  LDL-cholesterol  HDL-cholesterol | 6 weeks | Zinc p= 0.03  FPG p=0.005  insulin p=0.003  HOMA-IR p=0.001  HOMA-B p=0.01  QUICKI p=0.004  triglycerides p=0.01  VLDL-cholesterol p=0.01  Total cholesterol p=0.39  LDL-cholesterol p=0.43  HDL-cholesterol p=0.22 |
| Anderson et al., 2001 | Tunez | CG: 29  EG: 27 | CG: 53.8 ± 1.88  EG: 51.5 ± 1.62  Patients with T2DM | CG: Placebo  EG: Supplementation of zinc | CG: Placebo capsules daily for 6 months  EG: Zinc gluconate (containing 30 mg zinc) daily for 6 months | BMI (kg/m2)  Fasting Glucose (mmol/L)  HbA1c (%)  Insulin (pmol/L)  Cortisol (nmol/L)  Total Cholesterol (mmol/L)  HDL Cholesterol (mmol/L)  Plasma Zn mmol/LCu  Plasma Cu mmol/L  Plasma Se mmol/L  Urine Cr (ng/mg Ct)  Urine Zn (mg/mg Ct)  TBARS (mmol/L)  Cu-Zn SOD  RBC (U/g Hb)  Se GPx  RBC (U/mg Hb) | 26 weeks | BMI p= -  Fasting Glucose p= -  HbA1c p>0.05  Insulin p= -  Cortisol p= -  Total Cholesterol p= -  HDL Cholesterol p= -  Plasma Zn p<0.05  Plasma Cu p= -  Plasma Se p= -  Urine Cr p= -  Urine Zn p= -  TBARS p<0.001  Cu-Zn SOD  RBC p= -  Se GPx = -  RBC p= - |
| Asghari et al., 2019 | Iran | CG: 30  EG: 30 | CG: 45.5 ± 5.4  EG: 46.2 ± 5.3  Patients with T2DM | CG: Placebo  EG: Supplementation of zinc | CG: placebo daily for 12 weeks  EG: 30mg zinc as zinc gluconate daily for 12 weeks | Adiponectin (μg/ml)  Zinc (μg/dl)  HbA1c (%)  Fasting Blood Glucose (mg/dl)  Insulin (μIU/ml)  HOMA-IR  Triglycerides (mg/dl)  HDL (mg/dl)  LDL (mg/dl)  Cholesterol (mg/dl) | 12 weeks | Adiponectin p= 0.359  Zinc p= < 0.001  HbA1c p= 0.81  Fasting Blood Glucose p= 0.398  Insulin p= 0.131  HOMA-IR p= 0.913  Triglyceride p= 0.723  HDL p= 0.039  LDL p= 0.446  Cholesterol p= 0.308 |
| Hamedifard et al., 2020 | Iran | CG: 28  EG: 27 | CG: 62.6 ± 10.8  EG: 61.7 ± 9.4  Patients suffering from T2DM with proven 2-and 3-vessel CHD | CG: Placebo  EG: Supplementation of zinc and magnesium | CG: placebo per day for 12 weeks  EG: 250 mg magnesium (magnesium oxide) and 150 mg zinc sulfate (containing 30 mg elementary zinc) daily for 12 weeks | Magnesium (mg/dL)  Zinc (μg/dL)  FPG (mg/dL)  Insulin (μIU/mL)  HOMA-IR  QUICKI  Triglycerides (mg/dL)  VLDL-cholesterol (mg/dL)  Total cholesterol (mg/dL)  LDL-cholesterol (mg/dL)  HDL-cholesterol (mg/dL)  Total−/HDL-cholesterol ratio  CRP (mg/L)  Total nitrite (μmol/L)  TAC (mmol/L)  GSH (μmol/L)  MDA (μmol/L)  BDI score  BAI score | 12 weeks | Magnesium p= < 0.001  Zinc p= < 0.001  FPG p=0.03  Insulin p=0.02  HOMA-IR p=0.06  QUICKI p=0.12  Triglycerides p=0.30  VLDL-cholesterol p=0.30  Total cholesterol p= 0.41  LDL-cholesterol p=0.97  HDL-cholesterol p=0.04  Total−/HDL-cholesterol ratio p=0.59  CRP p= < 0.001  Total nitrite p=0.003  TAC p=0.03  GSH p=0.12  MDA p=0.05  BDI score p=0.04  BAI score p=0.02 |
| Jamilian et al., 2019 | Iran | CG: 30  EG: 30 | CG: 29.1 ± 4.1  EG: 27.7 ± 4.0  Women with GDM. | CG: Placebo  EG: Supplementation of magnesium, zinc, calcium, and vitamin D | CG: Placebo twice a day for 6 weeks.  EG: 100 mg magnesium, 4mg zinc, 400 mg calcium, and 200 IU vitamin D supplements twice a day for 6 weeks. | FPG  Magnesium  Zinc  Calcium  25-OH-vitamin D  hs-CRP  Total nitrate  TAC  GSH  MDA | 6 weeks | FPG p=0.008  Magnesium p=0.002  Zinc p<0.001  Calcium p=0.001  25-OH-vitamin D p=0.001  hs-CRP p=0.01  Total nitrate p=0.76  TAC p=0.01  GSH p=0.32  MDA p=0.003 |
| Attia et al., 2022 | Australia | CG: 50  EG: 48 | CG: 63.6 (58.9, 67.2)  EG: 61.6 (54.4, 66.2)  Pre-diabetic Patients | CG: Placebo  EG: Supplementation of Zinc | CG: Placebo (Cellulose capsules) per day for 12 months  EG: 30 mg elemental zinc  gluconate per day for 12 months | HbA1c  Fasting blood  glucose level  Beta cell function (%B)  Insulin sensitivity (%S)  Insulin resistance (IR)  Total cholesterol  LDL  HDL  Triglycerides | 52 weeks | HbA1c p=0.3214  Fasting blood  glucose level p=0.1386  Beta cell function (%B) p=0.6521  Insulin sensitivity (%S)  p=0.0381  Insulin resistance (IR)  p=0.0463  Total cholesterol p=0.5367  LDL p=0.6797  HDL p=0.9158  Triglycerides p=0.7774 |
| Nazem et al.,2022 | Iran | CG: 35  EG: 35 | CG: 40 - 65    EG: 40 - 65  Patients with T2DM | CG: Placebo  EG: Supplementation of zinc | CG: Placebo (Avicel capsules) daily for 8 weeks  EG: 50mg/day (2 capsules x 25 mg) of zinc gluconate daily for 8 weeks | FBG (mg/dL)  HbA1c (%)  Insulin (µU/mL)  HOMA-IR  Serum zinc  Weight (kg)  BMI (Kg/m2)  Serum MDA (nM/ml)  Serum levels Nitrotyrosine (ng/ml)  Serum TAC (mM)  Serum TOS (µM) | 8 weeks | FBG p<0.001  HbA1c p<0.001  Insulin p=0.02  HOMA-IR p<0.001  Serum zinc p<0.001  Weight p<0.001  BMI p<0.001  Serum MDA p<0.001  Serum levels of Nitrotyrosine p=0.002  Serum TAC p<0.001  Serum TOS p<0.001 |
| Pérez et al., 2018 | Chile | CG: 15  EG: 13 | CG: 56.0 [51.0–62.0]  EG: 55.0 [53.5–60.0]  Patients with T2DM | CG: Placebo  EG: Supplementation of zinc | CG: Placebo daily for 1 year  EG: 30 mg/day of zinc in the form of one capsule containing zinc sulfate for 1 year | Weight (kg)  BMI (kg/m2)  Fasting blood glucose (mg/dL)  HbA1c (%) (mmol/mol)  HOMA-IR  Triglycerides (mg/dL)  Total cholesterol (mg/dL)  HDL-cholesterol (mg/dL)  LDL-cholesterol (mg/dL)  VLDL-cholesterol (mg/dL)  Plasma glucagon concentration (pg/mL)  Glucagon/glucose ratio (pg/mg)FBS  Glucagon/insulin ratio (pg/μIU) | 52 weeks | Weight p=0.089  BMI p=0.002  Fasting blood glucose p=0.490  HbA1c p=0.419  HOMA-IR p=0.461  Triglycerides p=0.811  Total cholesterol p=0.062  HDL-cholesterol p=0.213  LDL-cholesterol p=0.122  VLDL-cholesterol p=0.809  Plasma glucagon concentration p=0.222  Glucagon/glucose ratio p=189  Glucagon/insulin ratio p=0.908 |
| Matter et al., 2019 | Egypt | CG: 39  EG: 38 | CG: 16.1 ± 1.4  EG: 16.4 ± 1.4  Pediatric β-TM patients complicated with DM | CG: Placebo  EG: Supplementation of zinc | CG: Placebo daily for 12 weeks  EG: 40 mg/day zinc gluconate (2 tablets of 20mg) for 12 weeks | FBG (mg/dL)  Fructosamine (mg/dL)  Fasting C-peptide (ng/mL)  HOMA-IR  BMI z-score  Systolic BP z-score    Diastolic BP z-score  WBC count (x 109/L)  Hemoglobin (g/dL)  LDH (IU/L)  Serum ferritin (µg/L)  Insulin dose (IU/Kg/day)  Serum creatinine (mg/dL)  UACR (mg/g creatinine)  Serum zinc (μg/dL) | 12 weeks | FBG p<0.001  Fructosamine p<0.001  Fasting C-peptide p=0.011  HOMA-IR p=0.002  BMI z-score p=519  Systolic BP z-score p=0.566    Diastolic BP z-score p=0.506  WBC count p<0.001  Hemoglobin p=0.010  LDH p=0.001  Serum ferritin p=0.029  Insulin dose p=0.179  Serum creatinine p=0.608  UACR p<0.001  Serum zinc p<0.001 |
| Momen-Heravi et al.,2017 | Iran | CG:30  EG:30 | CG: 60.0±10.0  EG: 58.3±8.6  Patients with grade 3 diabetic foot ulcer | CG: Placebo  EG: Supplementation of zinc | CG: Placebo daily for 12 weeks  EG: 220 mg zinc sulfate supplements (containing 50 mg elemental zinc) daily for 12 weeks | Ulcer length (cm)  Ulcer width (cm)  Ulcer depth (cm)  Zinc (mg/dL)    FPG (mg/dL)  Insulin ( IU/mL)    HOMA-IR  HOMA-B    QUICKI  HbA1c (%)  Triglycerides (mg/dL)  VLDL cholesterol (mg/dL)    Total cholesterol (mg/dL)  LDL cholesterol (mg/dL)  HDL cholesterol (mg/dL)  Total /HDLcholesterol ratio    hs-CRP (µg/mL)  ESR (mm/h)  NO (mol/L)  TAC (mmol/L)    GSH (µmol/L)  MDA (µmol/L) | 12 weeks | Ulcer length p=0.02  Ulcer width p=0.02  Ulcer depth p=0.04  Zinc p<0.001  FPG p=0.008  Insulin p=0.007  HOMA-IR p=0.005  HOMA-B p=0.07  QUICKI p=0.004  HbA1c p=0.01  Triglycerides p=0.27  VLDL cholesterol p=0.27    Total cholesterol p=0.24  LDLcholesterol p=0.10    HDLcholesterol p=0.01    Total /HDLcholesterol ratio p=0.01    hs-CRP p=0.02    ESR p=0.04  NO p<0.001  TAC p=0.003    GSH p=0.001    MDA p=0.03 |
| Karamali et al.,2016 | Iran | CG: 25  EG: 25 | CG: 29.3 ± 3.8  EG: 29.9 ± 5.0  Women with GDM | CG: Placebo  EG: Supplementation of zinc | CG: Placebo for 6 weeks  EG: 223mg zinc gluconate (containing 30mg zinc) for 6 weeks | Zinc (mg/dL)  hs-CRP (ng/mL)  NO (µU/mL)  TAC (mmol/L)  GSH (µU/mL)  MDA (µU/mL) | 6 weeks | Zinc p=0.41  hs-CRP p=0.01  NO p=0.83  TAC p=0.03  GSH p=0.84  MDA p=0.31 |
| Karandish et al., 2021 | Iran | CG: 21  EG: 21 | CG: 34.19 ± 7.03  EG: 34.48 ± 6.45  Effect of curcumin and zinc co- supplementation | CG: Placebo  EG: Supplementation of zinc and curcumin | CG: Placebo for 90 days  EG: Curcumin (500 mg) and Zinc (30 mg) | FPG (mg/dl)  2-hpp (mg/dl)  HbA₁C (%)  Insulin (µU/mL)  β-Cell function (%)  Insulin sensitivity (%)  Insulin resistance | 12 weeks | FPG p=0.007  2-hpp p= 0.004  HbA₁C p=0.02  Insulin p=0.001  β-Cell function p=0.82  Insulin sensitivity p=0.001  Insulin resistance p<0.001) |
| Nazem et al., 2019 | Iran | CG: 35  EG: 35 | CG: 54.34 ± 7.18  EG: 53.28 ± 7.35  Overweight participants with T2DM of at least five years | CG: Placebo  EG: Supplementation of zinc | CG: Placebo (Avicel capsule, Sigma) twice daily  EG: 25 mg zinc capsule twice daily | FBG  HbA1c  Insulin  HOMA-IR  Triglycerides  Total cholesterol  LDL-C    HDL-C | 8 weeks | FBG p < 0.001  HbA1c p < 0.001  Insulin p = 0.02  HOMA-IR p < 0.001  Triglycerides p = 0.01  Total cholesterol p = 0.04  LDL-C p = 0.01    HDL-C p < 0.001 |
| Shidfar et al., 2010 | Iran | CG: 23  EG: 25 | CG: 13.56 ± 3.55  EG: 13.16 3.48  DMTI patients | CG: Placebo  EG: Supplementation of zinc and vitamin A | CG: 10 ml Placebo syrup/day and a placebo tablet for 3 months.  EG: 10 ml zinc syrup containing 10 mg elemental zinc/day and one-half of a 25,000 IU vitamin A tablet every other day, for 3 months | Apoprotein B  Apoprotein A-I  FBS  Insulin  Apoprotein B/apoprotein A-I ratio | 13 weeks | Apoprotein B p<0.0001  Apoprotein A-I p< 0.0001  FBS p=-  Insulin p=-  Apoprotein B/apoprotein A-I ratio p=0.02 |
| Islam et al., 2016 | Bangladesh | CG: 27  EG: 28 | CG: 45.5 ± 10.4  EG: 42.1 ± 7.0  Patients with pre-diabetes | CG: Placebo  EG: Supplementation of zinc sulphate | CG: Placebo for six  months  EG: 30mg zinc sulphate dispersible tablet, once daily for six months.  - | FBG (mmol/l)  Blood glucose (mmol/l)  Serum zinc (ppb)  Serum insulin (μU/ml)  Triglyceride (mg/dl)  HDL (mg/dl)  LDL (mg/dl)  C reactive protein (mg/l)  Beta cell  function (%B)  Insulin  sensitivity (%S)  Insulin  resistance | 26 weeks | FBG p= 0.4  Blood glucose p= 0.2  Serum zinc p= 0.3  Serum insulin p= 0.09  Triglyceride p= <0.001  HDL p= 0.01  LDL p= 0.003  C reactive protein p= 0.4  Beta cell  function p= <0.001  Insulin  sensitivity p= 0.01  Insulin  resistance p= 0.001 |
| Hosseini et al., 2022 | Iran | CG: 22  EG: 21 | CG: 54.1 ± 5.4  EG: 55.6 ± 7.3  DHPs with Zn-deficiency | CG: Placebo  EG: Supplementation of zinc sulphate | CG: placebo capsule  (220 mg/day corn starch) for 8 weeks  EG: 220 mg/day ZnSO4  capsules (containing 50 mg Zn) for 8 weeks | BMI (kg/m2)  Weight (kg)  Zinc (μg/dL)  BUN (mg/dL)  Cr (mg/dL)  Copeptin (pg/mL)  Hs-CRP (mg/L)  FBG (mg/dL)  Insulin (μIU/ml)  HOMA-IR  QUICKI | 8 weeks | BMI p < 0.001  Weight p < 0.001  Zinc p < 0.001  BUN p < 0.001  Cr p < 0.001  Copeptin p= 0.02  Hs-CRP p < 0.001  FBG p< 0.001  Insulin p= 0.55  HOMA-IR p= 0.6  QUICKI p= 0.57 |
| Roussel et al; 2003 | UK | CG: 54  EG: 54 | CG: 55.5 ± 1.43  EG: 51.5 ± 1.62  Zinc supplementation in persons with type 2 DM | CG: Placebo  EG: Supplementation of zinc | CG: Placebo for six months  EG: 30 mg/day of Zinc as Zinc gluconate | Weight (kg)  BMI (kg/m²)  Fasting Glucose (mmol/L)  HbA1c (%)  Insulin (pmol/L)  Cortisol (nmol/L)  Total Cholesterol (mmol/L)  HDL Cholesterol (mmol/L) | 26 weeks | Weight p = >0.05  BMI p = >0.05  Fasting Glucose p = >0.05  HbA1c p = >0.05  Insulin p = >0.05  Cortisol p = >0.05  Total Cholesterol p = >0.05  HDL Cholesterol p = >0.05 |
| Seet et al; 2011 | Singapore | CG: 20  EG: 20 | CG: 55 ± 8  EG: 57 ± 9  Effects of supplementation of zinc in patients with type 2 diabetes | CG: Placebo  EG: Supplementation of zinc | CG: Placebo for 3 months  EG: 240 mg/day of zinc as zinc gluconate | Arachidonate (µg/ml)  Total F₂-IsoPs (ng/ml)  Esterified F₂-IsoPs (ng/ml)  Free F₂-IsoPs (ng/ml)  Total F₂-IsoPs/AA (pg/µg)  Esterified F₂-IsoPs/AA (pg/µg)  Urinary F₂-IsoPs (ng/mg Cr)  Urinary 2,3-dinor-5,6-dihydro F₂-IsoPs (ng/mg Cr)  Urinary 2,3-dinor F₂-IsoPs (ng/mg Cr)  Total HETEs (ng/ml)  Esterified HETEs (ng/ml)  Free HETEs (ng/ml)  Total HETEs/AA (ng/µg)  Esterified HETEs/AA (ng/µg)  Urinary HETEs (ng/mg Cr)  Docosahexaenoate (µg/ml)  Total neuroprostanes (ng/ml)  Esterified neuroprostanes (ng/ml)  Free neuroprostanes (ng/ml)  Total neuroprostanes/DHA (pg/µg)  Esterified neuroprostanes/DHA (pg/µg)  7β-Hydroxycholesterol (ng/mg cholesterol)  7-Ketocholesterol (ng/mg cholesterol)  7α-Hydroxycholesterol (ng/mg cholesterol)  24-Hydroxycholesterol (ng/mg cholesterol)  27-Hydroxycholesterol (ng/mg cholesterol)  Allantoin (µM)  Urate (µM)  Allantoin/urate  Nitrite (µM)  Nitrate (µM)  Total nitrate + nitrite  SBP (mm Hg)  DBP (mm Hg)  Augmentation index (%)  Pulse wave velocity (m/s) | 13 weeks | Arachidonate p= 0.74)  Total F₂-IsoPs p= 0.10  Esterified F₂-IsoPs p= 0.11  Free F₂-IsoPs p= 0.21  Total F₂-IsoPs/AA p= 0.10  Esterified F₂-IsoPs/AA  p= 0.10  Urinary F₂-IsoPs p= 0.83  Urinary  2,3-dinor-5,6-dihydro F₂-IsoPs p= 0.80  Urinary 2,3-dinor F₂-IsoPs  p= 0.61  Total HETEs p= 0.60  Esterified HETEs p= 0.59  Free HETEs p= 0.16  Total HETEs/AA p= 0.26  Esterified HETEs/AA p= 0.26  Urinary HETEs p= 0.12  Docosahexaenoate p= 0.97  Total neuroprostanes  p= 0.83  Esterified neuroprostanes  p= 0.77  Free neuroprostanes  p= 0.37  Total neuroprostanes/DHA  p= 0.77  Esterified neuroprostanes/DHA  p= 0.59  7β-Hydroxycholesterol  p= 0.38  7-Ketocholesterol  p= 0.30  7α-Hydroxycholesterol  p= 0.66  24-Hydroxycholesterol  p= 0.96  27-Hydroxycholesterol  p= 0.70  Allantoin p= 0.77  Urate p= 0.97  -Allantoin/urate p= 0.91  Nitrite p= 0.17  Nitrate p= 0.28  Total nitrate + nitrite p= 0.30  SBP p= 0.33  DBP p= 0.33  Augmentation index p= 0.06  Pulse wave velocity p= 0.14 |

**GDM:** Gestational diabetes mellitus**; DM:**Diabetes mellitus; **β-TM**:β-Thalassemia Major; **SD:** Standard deviation; **EG:** Experimental group; **CG:** Control group; **FPG**: fasting plasma glucose; **FBG**: fasting blood glucose; **T2DM**: Type 2 Diabetes Mellitus; **VAS:** Visual Analog Scale; **ᵇ**: Mann-Whitney U test for not normally distributed parameters; **NT**: Nitrotyrosine; **TOS**: Total Oxidant Status;**TAC**: Total Antioxidant Capacity ;**MDA:** malondialdehyde; **BMI**: Body Mass Index; **CHD:** Coronary Heart Disease; **QUICKI:** quantitative insulin sensitivity check index; **BDI score:** Beck Depression Inventory; **BAI:** Beck Anxiety Inventory; **GSH:** total glutathione; **HbA1c**: Glycosylated hemoglobin; **HOMA-IR**: Homeostasis Model Assessment – Insulin Resistance; **HOMA-B**: Homeostasis Model Assessment of β-cell function; **SD:** Standard deviation; **TBARS:**Thiobarbituric Acid Reactive Substances; **CRP:** C-reactive protein; **OGTT:** Oral glucose tolerance test; **WBC:** white blood cell; **UACR:** Urine Albumin-Creatinine Ratio;**LDL:** low density lipoproteins; **HDL:** high lipoproteins density; **VLDL:** very low density lipoprotein; **SBP**: Systolic Blood Pressure; **DBP**: Diastolic Blood Pressure; **NO**: nitric oxide; **2-hpp:** 2 Hour Postprandial Blood Glucose; **HDL-C:** high-density lipoprotein-cholesterol, **LDL-C:** low-density lipoprotein-cholesterol; **DMTI:** Diabetes mellitus tipo 1; **DHPs**: diabetic hemodialysis patients; **FBS:** Fasting Blood Sugar;

**Table S4.** Summary of Findings (SoF) and GRADE certainty of evidence for zinc supplementation in diabetic individuals.

| **Certainty assessment** | | | | | | | **№ of individuals** | | **Effect** | | **Quality of evidence (GRADE)** | **Importance** |
| --- | --- | --- | --- | --- | --- | --- | --- | --- | --- | --- | --- | --- |
| **№ of studies** | **Study design** | **Risk of bias** | **Inconsistency** | **Indirectness** | **Imprecision** | **Other considerations** | **Supplementation With zinc Mg** | **Control group** | **Relative (95% CI)** | **MD (95% CI)** |  |  |
| **Plasma zinc** | | | | | | | | | | | | |
| 9 | RCT | Not serious | Serious | Not serious | Not serious | None | 265 | 266 | - | 7.80 (4.33 to 11.26) | ⨁⨁⨁◯ moderate | CRITICAL |
| **Fasting plasma glucose (FPG)** | | | | | | | | | | | | |
| 5 | RCT | Not serious | serious | Not serious | Serious | None | 137 | 137 | - | −4.35 ( −10.80 to 2.11) | ⨁⨁◯◯ Low | CRITICAL |
| **Fasting blood glucose (FBG)** | | | | | | | | | | | | |
| 5 | RCT | Not serious | Serious | Not serious | Serious | None | 170 | 171 | - | −0.61 ( −1.83 to 0.60) | ⨁⨁◯◯ Low | CRITICAL |
| **Serum insulin** | | | | | | | | | | | | |
| 11 | RCT | Not serious | Serious | Not serious | Not serious | None | 300 | 302 | - | −2.50 ( −4.69 to −0.31) | ⨁⨁⨁◯ moderate | CRITICAL |

**HOMA-IR level**

| 7 | RCT | Not serious | Serious | Not serious | Not serious | None | 185 | 189 | - | −1.10 ( −2.05 to −0.15) | ⨁⨁⨁◯ Moderate | CRITICAL |
| --- | --- | --- | --- | --- | --- | --- | --- | --- | --- | --- | --- | --- |

**HOMA-B level**

| 2 | RCT | Not serious | Not serious | Not serious | Not serious | None | 59 | 59 | - | −23.78 ( −30.96 to −16.60) | ⨁⨁◯◯ Low | CRITICAL |
| --- | --- | --- | --- | --- | --- | --- | --- | --- | --- | --- | --- | --- |

**NO (Nitric oxide) level**

| **2** | **RCT** | **Not serious** | **Serious** | **Not serious** | **Serious** | **None** | **59** | **59** | **-** | **−2.02 ( −4.90 to 0.87)** | **⨁⨁◯◯ Low** | **CRITICAL** |
| --- | --- | --- | --- | --- | --- | --- | --- | --- | --- | --- | --- | --- |

**QUICKI level**

| **2** | **RCT** | **Not serious** | **Serious** | **Not serious** | **Serious** | **None** | **55** | **55** | **-** | **1.36 ( −4.14 to 6.87)** | **⨁◯◯◯**  **Very Low** | **IMPORTANT** |
| --- | --- | --- | --- | --- | --- | --- | --- | --- | --- | --- | --- | --- |

**Triglycerides level**

| 7 | RCT | Not serious | Serious | Not serious | Serious | None | 192 | 194 | - | −5.37 (−29.55 to 18.81) | ⨁◯◯◯ Very low | IMPORTANT |
| --- | --- | --- | --- | --- | --- | --- | --- | --- | --- | --- | --- | --- |

**LDL-cholesterol level**

| 7 | RCT | Not serious | Not serious | Not serious | Not serious | None | 192 | 194 | - | 3.46 (1.48 to 5.43) | ⨁⨁⨁◯ Moderate | IMPORTANT |
| --- | --- | --- | --- | --- | --- | --- | --- | --- | --- | --- | --- | --- |

**HDL-cholesterol**

| 7 | RCT | Serious | Serious | Not serious | Serious | None | 192 | 194 | - | 2.67 (−0.24 to 5.59) | ⨁◯◯◯ Very low | IMPORTANT |
| --- | --- | --- | --- | --- | --- | --- | --- | --- | --- | --- | --- | --- |

**Total/HDL-cholesterol ratio**

| 2 | RCT | Not serious | Serious | Not serious | Serious | None | 57 | 58 | - | −0.24 (−0.73 to 0.25) | ⨁◯◯◯  Very Low | IMPORTANT |
| --- | --- | --- | --- | --- | --- | --- | --- | --- | --- | --- | --- | --- |

**VLDL-cholesterol**

| 4 | RCT | Serious | Serious | Not serious | Serious | None | 99 | 102 | - | −2.53 (−7.02 to 1.96) | ⨁◯◯◯ Very low | IMPORTANT |
| --- | --- | --- | --- | --- | --- | --- | --- | --- | --- | --- | --- | --- |

**Total cholesterol**

| 6 | RCT | Not serious | Not serious | Not serious | Serious | None | 164 | 167 | - | −5.70 (−7.50 to −3.89) | ⨁⨁⨁⨁ High | IMPORTANT |
| --- | --- | --- | --- | --- | --- | --- | --- | --- | --- | --- | --- | --- |

**HbA1c**

| 4 | RCT | Serious | Serious | Not serious | Serious | None | 116 | 115 | - | 0.04 ( −0.26 to 0.35) | ⨁◯◯◯  Very Low | CRITICAL |
| --- | --- | --- | --- | --- | --- | --- | --- | --- | --- | --- | --- | --- |

**BMI**

| 4 | RCT | Not serious | Not serious | Not serious | Serious | None | 108 | 113 | - | 0.85 ( 0.57 to 1.14) | ⨁⨁⨁◯ Moderate | IMPORTANT |
| --- | --- | --- | --- | --- | --- | --- | --- | --- | --- | --- | --- | --- |

**Magnesium**

| 2 | RCT | Not serious | Not serious | Not serious | Not serious | None | 57 | 58 | - | 0.15 (0.08 to 0.22) | ⨁⨁⨁⨁ High | CRITICAL |
| --- | --- | --- | --- | --- | --- | --- | --- | --- | --- | --- | --- | --- |

**CRP/hs-CRP**

| 5 | RCT | Serious | Not serious | serious | Not serious | None | 133 | 138 | - | −0.91 ( −1.43 to −0.38) | ⨁⨁⨁◯ moderate | CRITICAL |
| --- | --- | --- | --- | --- | --- | --- | --- | --- | --- | --- | --- | --- |

**TAC (total antioxidant capacity)**

| 4 | RCT | Serious | Not serious | serious | Not serious | None | 112 | 113 | - | 1.79 (0.68 to 2.91) | ⨁⨁⨁◯ moderate | CRITICAL |
| --- | --- | --- | --- | --- | --- | --- | --- | --- | --- | --- | --- | --- |

**GSH (total glutathione)**

| 4 | RCT | Serious | Serious | Not serious | Serious | None | 112 | 113 | - | 0.20 ( −0.21 to 0.61) | ⨁⨁◯◯ Low | IMPORTANT |
| --- | --- | --- | --- | --- | --- | --- | --- | --- | --- | --- | --- | --- |

**MDA/Serum MDA (malondialdehyde)**

| 4 | RCT | Serious | Not serious | serious | Serious | None | 112 | 113 | - | −0.76 (−1.34 to −0.18) | ⨁⨁⨁◯ moderate | CRITICAL |
| --- | --- | --- | --- | --- | --- | --- | --- | --- | --- | --- | --- | --- |

**Weight**

| 2 | RCT | Not serious | Not serious | serious | Not serious | None | 34 | 40 | - | 2.38 (1.40 to 3.35) | ⨁⨁◯◯ Low | IMPORTANT |
| --- | --- | --- | --- | --- | --- | --- | --- | --- | --- | --- | --- | --- |

**SBP (systolic blood pressure)**

| 2 | RCT | Not serious | Serious | Not serious | Serious | None | 59 | 58 | - | −1.85 ( −10.14 to 6.44) | ⨁⨁◯◯ Low | IMPORTANT |
| --- | --- | --- | --- | --- | --- | --- | --- | --- | --- | --- | --- | --- |

**Insulin sensitivity**

| 2 | RCT | Not serious | Not serious | Serious | Serious | None | 49 | 47 | - | 1.78 ( 0.46 to 3.09) | ⨁⨁⨁◯ moderate | CRITICAL |
| --- | --- | --- | --- | --- | --- | --- | --- | --- | --- | --- | --- | --- |

**Insulin resistance**

| 2 | RCT | Not serious | Not serious | Not serious | Serious | None | 49 | 47 | - | −0.04 ( −0.06 to −0.02) | ⨁⨁⨁◯ Moderate | CRITICAL |
| --- | --- | --- | --- | --- | --- | --- | --- | --- | --- | --- | --- | --- |

**Beta Cell function**

| 2 | RCT | Not serious | Not serious | serious | Not serious | None | 49 | 47 | - | 8.08 (4.98 to 11.18) | ⨁⨁⨁◯ moderate | CRITICAL |
| --- | --- | --- | --- | --- | --- | --- | --- | --- | --- | --- | --- | --- |

**Quality of evidence: High:** The research provides a very good indication of the likely effect, and the probability that the true effect is substantially different is low. **Moderate:** The research provides a good indication of the likely effect, but the probability that the true effect is substantially different is moderate. **Low:** The research provides some indication of the probable effect, but the probability that the true effect is substantially different is high. **Very low:** The research does not provide a reliable estimate of the probable effect, and the probability that the true effect is substantially different is very high. In the GRADE system, randomized clinical trials start at high certainty, whereas observational studies start at low certainty, before considering potential downgrading or upgrading factors. Downgrading criteria: The GRADE approach considers four domains for potentially downgrading the certainty of evidence. Evaluation begins with the study design (RCTs or observational studies), and evidence is downgraded by one level if: (1) study limitations (risk of bias) are detected, particularly when most included studies (>50%) have high risk of bias; (2) inconsistency is present, typically assessed through heterogeneity statistics, and downgraded when I² exceeds the accepted threshold (I² > 40%); (3) indirectness occurs if the evidence does not directly answer the research question (e.g., due to differences in population, intervention, comparator, or outcomes); or (4) imprecision, which is observed when confidence intervals are wide, cross the line of no effect, or when the total sample size is small (commonly n < 400 for continuous outcomes), limiting the certainty about the magnitude or direction of the effect.

**Table S5:** Complete moderator analyses.

| Outcome | Moderator | B | 95% LLCI | 95% ULCI | P- value |
| --- | --- | --- | --- | --- | --- |
| Zinc | Mean age (Approx) | 0.00 | -0.01 | 0.00 | 0.4935 |
| Zinc | Supplementation weeks | 0.20 | -0.20 | 0.60 | 0.3254 |
| Zinc | Daily dose (mg) | 0.01 | -0.03 | 0.04 | 0.6566 |
| Zinc | Proportion of women | -9.03 | -17.82 | -0.23 | 0.0444 |
| LDL cholestorol | Mean age (Approx) | 0.00 | 0.00 | 0.00 | 0.0126 |
| LDL cholestorol | Supplementation weeks | -0.01 | -0.08 | 0.06 | 0.7741 |
| LDL cholestorol | Daily dose (mg) | 0.00 | -0.01 | 0.01 | 0.7968 |
| LDL cholestorol | Proportion of women | -0.63 | -4.73 | 3.47 | 0.7269 |
| HDL cholestorol | Mean age (Approx) | 0.00 | 0.00 | 0.00 | 0.0116 |
| HDL cholestorol | Supplementation weeks | -0.02 | -0.04 | 0.00 | 0.0451 |
| HDL cholestorol | Daily dose (mg) | 0.00 | 0.00 | 0.01 | 0.1133 |
| HDL cholestorol | Proportion of women | 0.47 | -0.76 | 1.69 | 0.4549 |
| VLDL cholesterol | Mean age (Approx) | 0.00 | 0.00 | 0.00 | 0.0000 |
| VLDL cholesterol | Supplementation weeks | 0.03 | -0.07 | 0.13 | 0.5725 |
| VLDL cholesterol | Daily dose (mg) | -0.01 | -0.03 | 0.01 | 0.44.94 |
| VLDL cholesterol | Proportion of women | -2.45 | -8.32 | 3.43 | 0.4142 |
| Total cholesterol | Mean age (Approx) | 0.00 | 0.00 | 0.00 | 0.0000 |
| Total cholesterol | Supplementation weeks | 0.00 | -0.03 | 0.04 | 0.8212 |
| Total cholesterol | Daily dose (mg) | 0.00 | -0.01 | 0.00 | 0.1990 |
| Total cholesterol | Proportion of women | -0.97 | -2.68 | 0.75 | 0.2691 |
| Triglycerides | Mean age (Approx) | -0.02 | -0.03 | -0.01 | 0.0037 |
| Triglycerides | Supplementation weeks | 0.32 | -0.77 | 1.42 | 0.5651 |
| Triglycerides | Daily dose (mg) | -0.12 | -0.26 | 0.03 | 0.1242 |
| Triglycerides | Proportion of women | -32.59 | -85.93 | 20.74 | 0.2310 |
| TAC | Mean age (Approx) | 0.00 | 0.00 | 0.00 | 0.1106 |
| TAC | Supplementation weeks | 0.07 | -0.48 | 0.62 | 0.8005 |
| TAC | Daily dose (mg) | 0.00 | -0.02 | 0.02 | 0.8001 |
| TAC | Proportion of women | 1.54 | -2.73 | 5.82 | 0.8755 |
| QUICKI | Mean age (Approx) | 0.00 | 0.00 | 0.00 | 0.4788 |
| QUICKI | Supplementation weeks | 0.00 | 0.00 | 0.00 | 0.5605 |
| QUICKI | Daily dose (mg) | 0.00 | 0.00 | 0.00 | 0.6705 |
| QUICKI | Proportion of women | -0.01 | -0.03 | 0.00 | 0.4490 |
| GSH | Mean age (Approx) | 0.00 | 0.00 | 0.00 | 0.1300 |
| GSH | Supplementation weeks | 0.06 | -0.12 | 0.24 | 0.5379 |
| GSH | Daily dose (mg) | 0.00 | 0.00 | 0.01 | 0.2134 |
| GSH | Proportion of women | 1.02 | -1.88 | -0.16 | 0.0204 |
| BMI | Mean age (Approx) | 0.00 | 0.00 | 0.00 | 0.0202 |
| BMI | Supplementation weeks | -0.01 | -0.06 | 0.36 | 0.2514 |
| BMI | Daily dose (mg) | 0.01 | 0.00 | 0.01 | 0.5117 |
| hs-CRP | Mean age (Approx) | 0.00 | 0.00 | 0.00 | 0.0001 |
| hs-CRP | Supplementation weeks | 0.08 | -0.20 | 0.36 | 0.0064 |
| hs-CRP | Daily dose (mg) | 0.00 | -0.01 | 0.01 | 0.5907 |
| hs-CRP | Proportion of women | 0.77 | -1.46 | 3.01 | 0.3680 |
| Serum MDA | Mean age (Approx) | 0.00 | 0.00 | 0.00 | 0.4960 |
| Serum MDA | Supplementation weeks | 0.12 | -0.12 | 0.36 | 0.0011 |
| Serum MDA | Daily dose (mg) | 0.00 | 0.00 | 0.01 | 0.3137 |
| Serum MDA | Proportion of women | -0.53 | -2.93 | 1.87 | 0.2658 |
| Serum Insuline | Mean age (Approx) | 0.00 | 0.00 | 0.00 | 0.6652 |
| Serum Insuline | Supplementation weeks | -0.02 | -0.15 | 0.11 | 0.7307 |
| Serum Insuline | Daily dose (mg) | 0.00 | -0.01 | 0.01 | 0.4703 |
| Serum Insuline | Proportion of women | -2.99 | -6.88 | 0.89 | 0.1312 |
| HOMA-IR | Mean age (Approx) | 0.08 | 0.00 | 0.16 | 0.0420 |
| HOMA-IR | Supplementation weeks | 0.04 | -0.05 | 0.12 | 0.3765 |
| HOMA-IR | Daily dose (mg) | -0.01 | -0.02 | 0.00 | 0.0944 |
| HOMA-IR | Proportion of women | -4.31 | -8.68 | 0.06 | 0.0531 |
| Hba1c | Mean age (Approx) | 0.00 | 0.00 | 0.00 | 0.1677 |
| Hba1c | Supplementation weeks | -0.11 | -0.52 | 0.30 | 0.5971 |
| Hba1c | Daily dose (mg) | -0.01 | -0.01 | 0.00 | 0.0734 |
| Hba1c | Proportion of women | 4.36 | 2.10 | 6.61 | 0.0002 |
| FPG | Mean age (Approx) | 0.00 | 0.00 | 0.00 | 0.0921 |
| FPG | Supplementation weeks | 0.17 | -0.38 | 0.72 | 0.5419 |
| FPG | Daily dose (mg) | -0.01 | -0.02 | 0.00 | 0.1139 |
| FPG | Proportion of women | -0.79 | -6.81 | 5.24 | 0.7979 |
| FBG | Mean age (Approx) | 0.00 | 0.00 | 0.00 | 0.1912 |
| FBG | Supplementation weeks | 0.00 | -0.07 | 0.07 | 0.995 |
| FBG | Daily dose (mg) | -0.01 | -0.02 | 0.01 | 0.2714 |
| FBG | Proportion of women | 0.21 | -5.212 | 5.64 | 0.9388 |
